# Antibiofilm and Antimicrobial Potentials of Novel Synthesized Sulfur Camphor Derivatives

**DOI:** 10.3390/ijms252010895

**Published:** 2024-10-10

**Authors:** Anna Duda-Madej, Szymon Viscardi, Katarzyna Pacyga, Robert Kupczyński, Wanda Mączka, Małgorzata Grabarczyk, Paweł Pacyga, Ewa Topola, Michał Ostrówka, Jacek Bania, Antoni Szumny, Katarzyna Wińska

**Affiliations:** 1 Department of Microbiology, Faculty of Medicine, Wroclaw Medical University, Chałubińskiego 4, 50-368 Wrocław, Poland; 2 Faculty of Medicine, Wroclaw Medical University, Ludwika Pasteura 1, 50-367 Wrocław, Poland; szymon.viscardi@student.umw.edu.pl (S.V.);; 3 Department of Environment Hygiene and Animal Welfare, Faculty of Biology and Animal Science, Wrocław University of Environmental and Life Sciences, 50-375 Wrocław, Polandrobert.kupczynski@upwr.edu.pl (R.K.); 4 Department of Food Chemistry and Biocatalysis, Faculty of Biotechnology and Food Science, Wrocław University of Environmental and Life Sciences, C.K. Norwida 25, 50-375 Wrocław, Poland; wanda.maczka@upwr.edu.pl (W.M.); antoni.szumny@upwr.edu.pl (A.S.); katarzyna.winska@upwr.edu.pl (K.W.); 5 Department of Thermodynamics and Renewable Energy Sources, Faculty of Mechanical and Power Engineering, Wrocław University of Science and Technology, 50-370 Wrocław, Poland; pawel.pacyga@pwr.edu.pl; 6 Faculty of Biotechnology, University of Wrocław, Fryderyka Joliot-Curie 14a, 50-137 Wrocław, Poland; michal.ostrowka2@uwr.edu.pl; 7 Department of Food Hygiene and Consumer Health Protection, Wrocław University of Environmental and Life Sciences, 50-375 Wrocław, Poland

**Keywords:** antibiofilm activity, antimicrobial activity, camphor, monoterpenoids, skin infections, sulfur derivatives

## Abstract

The question being posed by scientists around the world is how different chemical modifications of naturally occurring compounds will affect their antimicrobial properties. In the current study, sulfur derivatives of camphor containing a sulfur atom were tested to detect their antimicrobial and antibiofilm potentials. The new compounds were tested on eight Gram-positive strains (*S. aureus* (3 isolates), *S. epidermidis* (4 isolates), and *E. faecalis* (1 isolate)) and eight Gram-negative strains (*E. coli* (6 isolates), *A. baumannii* (1 isolate), and *P. aeruginosa* (1 isolate)). The ability of the strains to eradicate a biofilm was evaluated under standard stationary and flow-through conditions using the Bioflux system. Two synthesized compounds, namely *rac*-thiocamphor (**1a**) and (S, S)-(+)-thiocamphor (**2a**), exhibited an effect on the 24 h biofilm formed by the Gram-positive strains. Our results are an important contribution to the science of natural compounds and allow us to classify our sulfur derivatives of camphor as potential prophylactic agents in treating skin infections, antiseptics, and disinfectants. The Gram-negative strains were excluded from further stages of the tests due to their high activity (MIC ≥ 512 µg/mL). On the other hand, the compound with the strongest antimicrobial activity against the Gram-positive strains was **2a**, as it led led to a reductions in cell viability of 17–52% (for MIC), 37–66% (for 2MIC), and 40–94% (for 4MIC). In addition, the experimental retention index of thiocamphor was calculated for the first time.

## 1. Introduction

The skin is the largest organ of the body, comprising a complex network of multiple types of cells, and serves numerous vital functions (e.g., protection against environmental stresses) [1,2]. It is colonized by various microorganisms that form the skin microbiome, which plays a crucial role in maintaining the skin health [3,4] by sustaining the function of the epidermal barrier and immune homoeostasis, as well as preventing the growth of pathogenic bacteria and addressing the harmful consequences of microbial dysbiosis that lead to inflammation [3,5]. The bacteria of the human skin microbiome are classified into four groups, namely *Actinobacteria* (52%), *Firmicutes* (24%), *Proteobacteria* (16%), and *Bacteroidetes* (6%), and these represent a major part of the human skin microbiome (>90%). Among them, coagulase-negative *Staphylococcus* spp., particularly *Staphylococcus epidermidis*, anaerobic *Cutibacterium acnes*, *Corynebacterium* spp., *Micrococcus* spp., *Streptococcus* spp., and *Acinetobacter* spp., are the predominant species [6,7]. If the barrier function of the skin is interrupted by chronic skin disease, microorganisms on the skin surface have direct access to the skin, which leads to inflammation and an immune response [1,3].

Skin and soft tissue infections (SSTIs) are one of the most prevalent types of bacterial infection and constitute a major diagnostic and therapeutic challenge. Skin infections can be difficult to treat, and patients with complex SSTIs (cSSTIs) may need long-term hospitalization, in particular when the pathogens that caused the infection are resistant to drugs or host factors complicate the infection [3,8]. Noninfectious skin diseases can easily develop into bacterial infections over time. These infectious conditions can be caused by Gram-positive bacteria (including methicillin-sensitive and -resistant *Staphylococcus aureus;* MSSA and MRSA), as well as *Streptococcus* spp., which penetrate the skin tissues through skin lesions created by continued rubbing of the skin due to itching [3]. The general treatment of cSSTIs includes a combination of surgical debridement or drainage and antibiotic therapy. In addition, acute bacterial skin and skin structure infections (ABSSSIs) are common in a variety of healthcare facilities. Over the past two decades, MRSA has emerged as a significant cause of purulent skin infections with higher associated rates of complication (e.g., abscess), recurrence, and treatment failure, often leading to hospitalization. Until recently, the primary treatment for MRSA infections was vancomycin and teicoplanin glycopeptides [9]. However, concerns about the progressive development of the disease’s resistance and efficacy have highlighted the need to develop new active agents (co-trimoxazole and tetracycline) against Gram-positive bacteria [10]. Nevertheless, in some cases, large doses of antibiotics can cause systemic toxicity. Moreover, the development of new antibiotics has declined in recent years, and a limited number of companies remain active in these areas. At the same time, the number of antibiotic-resistant microorganisms has increased significantly due to the widespread overuse and misuse of antibiotics worldwide. This antimicrobial crisis continues to affect antibiotic therapies for both systemic and topical infections [11]. Skin infections caused by Gram-negative pathogens are also affected by this crisis. These are primarily infections associated with primary diseases such as diabetes (*osteomyelitis*), surgical site infections, bedsores, or burn wound infections. Particular clinical vigilance in this area is given to infections with the etiology of non-fermenting rods: *Pseudomonas aeruginosa, Acinetobacter baumannii*, and *Enterobacteriaceae*, especially *E. coli* [12,13]. These pathogens are often characterized by antibiotic resistance (e.g., KPC—*Klebsiella pneumoniae* carbapenemase, ESBL—extended-spectrum beta-lactamases) and are capable of forming biofilms that hinder their eradication [14].

It is worth mentioning that infectious bacteria can occur as single cells and as a biofilm. A biofilm is a structure that is often composed of different types of organisms surrounded by a layer of organic and inorganic substances that they produce. The microorganisms present in a biofilm are protected by the matrix and evade the host immune response, and are characterized by a high degree of antibiotic resistance. During infection, the host immune cells release enzymes, cytokines, and reactive oxygen species (ROS), which cause tissue destruction and increase the inflammatory process. Therefore, the healing process can be altered or completely inhibited [15,16]. It is also of the utmost importance to consider swimming motility in selecting applied therapies, as it allows bacteria to spread from persister cells in biofilm microcolonies and colonize other tissues [17].

Taking into account the existing issues posed by skin infections, the antimicrobial potential of nonconventional therapies has attracted renewed interest. In particular, the role of natural agents has drawn the attention of the scientific community and companies to the production of new bioproducts. In recent years, a significant increase in the utilization of essential oils and their active components has been observed [5,18,19,20]. The application of essential oils (EOs) in treating skin infections is highly promising as they regulate the quorum-sensing (QS) systems of pathogens and inhibit virulence expression. Interference with the QS system can potentially diminish multidrug resistance in bacteria [21]. As an example, camphor oil, which, due to its properties, can be used in the treatment of skin diseases of bacterial and fungal etiology, can be mentioned. It can support the treatment of acne, eczema, inflammation, ulcers and wounds, psoriasis, and fungal foot infections, as it exerts a local anaesthetic effect [5]. Furthermore, it is also effective against swelling and can remove keratinized epidermis. Therefore, it accelerates wound healing time and promotes faster regeneration of the damaged area. *Cinamonium camphora* oil has been shown to act as a potential antimicrobial agent and QS system inhibitor in the prevention of bacterial infection [21]. Complexes of camphor imine or camphor sulfonimine ligands were also studied [22]. All complexes studied showed higher activity against Gram-negative strains than against Gram-positive strains. However, the antibacterial activities of the Ag(I) camphor sulfonimine complexes were stronger than those of the camphor imine analogues [22].

One particularly promising finding is that Santos et al. proved that a flexible camphor diamond-like carbon coating on polyurethane leads to the inhibition (99% and 91% compared to polyurethane alone) of *Candida albicans* biofilm growth. The effect of camphor on the inhibition of the growth of the biofilm of pathogens on a potential material for use in the formation of vascular and urological catheters is an important premise for further research of this compound [23]. The thio-derivatives of camphor exhibit bactericidal properties, but their effect on the biofilms formed by Gram-positive and Gram-negative bacteria has not been determined.

The aim of our study was to synthesize sulfur compounds with camphor and to evaluate the impact of the sulfur replacement of ketone oxygen on the activity of camphor and its biofilm capacity. Our hypothesis was that thio-derivatives obtained from camphor essential oil would exhibit enhanced antimicrobial activity and the ability to effectively eradicate the bacterial biofilm structure, and, thus, could be used successfully in the treatment and prevention of skin infections.

## 2. Results and Discussion

**Figure 1:**
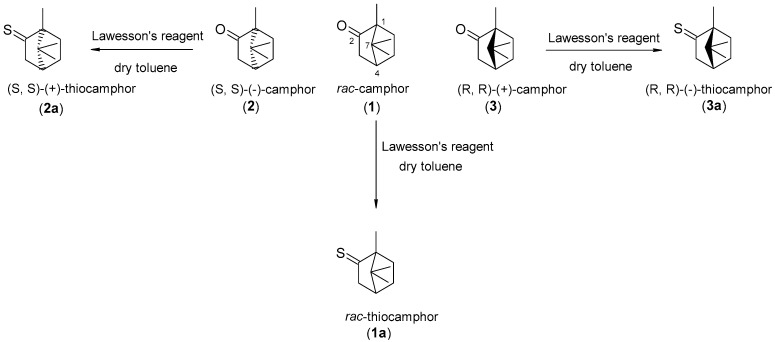
Synthesis of thio-analogs of camphor.

A number of review papers have been published to date on the antimicrobial activity of camphor and its derivatives [24,25,26,27,28,29], usually using (R)-(+)-camphor for the synthesis of these compounds. However, there is a lack of data on the effect of camphor’s stereochemistry on antimicrobial activity. For this reason, sulfur derivatives of both racemic camphor and its two isomers were obtained in the first stage of this study (Figure 1). The chemical reactions were carried out according to a well-known procedure [30], using Lawesson’s reagent in dry toluene under a nitrogen atmosphere. The progress of the reaction was controlled by gas chromatography, while the purity was additionally checked by NMR (Figures S1–S4). The samples with a purity of at least 98% were selected for further bioassays.

Retention indices are data that play a key role in the interpretation of compounds by gas chromatography, especially natural compounds. In this study, the experimental (logarithmic) value was measured for the first time using the standard type-5 column (5%-phenyl)-methylpolysiloxane. It is noteworthy that the value presented in the NIST23 database, based on an artificial intelligence algorithm, differs by about 50, which is unacceptable in a chromatographic study (Figures S5–S9).

### 2.1. Antimicrobial Activity

The attachment of a sulfur atom to the camphor molecule in different conformations improved the antimicrobial activity of the starting compound (Figure S10). This improvement can be observed primarily in Gram-positive bacteria. The highest difference in activity level was observed for compound pair 1 vs. **1a**. The introduction of sulfur into the starting mixture of racemic camphor contributed to a decrease in the MIC_50_ value by four orders for *S. aureus* RF 122, *S. epidermidis* S22, and *E. faecalis* ATCC 29212; by three orders for *S. epidermidis* 275lp; by two rows against *S. aureus* MRSAkj and *S. epidermidis* B145; and by one row for *S. aureus* ATCC 25923. A slightly weaker improvement in antimicrobial activity against compound 1 was observed for **2a**. A decrease in the MIC_50_ value by 2 orders for *S. aureus* RF 122 and MRSAkj and *S. epidermidis* B145 was also recorded for **2a**. In contrast, the MIC_50_ values were decreased by 1 order for *S. epidermidis* ATCC 12228 and S22. The weakest increase in antimicrobial activity was shown for 1 vs. 3a. The lowest MIC_50_ value recorded for the tested strains was 512 µg/mL. Moreover, against *S. epidermidis* 275lp, compound 1 showed better activity (128 µg/mL) than 3a after modifications were made (512 µg/mL).

Among the Gram-negative strains tested, a slight improvement in antimicrobial activity, from >512 µg/mL to 512 µg/mL, was observed for only *A. baumannii* ATCC 19606 (1 vs. **1a**), *E. coli* PCM 2427, and *P. aeruginosa* ATCC 27853 (1 vs. **2a**).

Among the sulfur derivatives, the highest antimicrobial activity against the tested Gram-positive strains was exhibited by the sulfur racemate derivative (**1a**). It showed the strongest activity against *S. epidermidis* 275lp (MIC_50_ = 16 µg/mL) and *E. faecalis* ATCC 29212 (MIC_50_ and MIC_90_ = 64 µg/mL). For *S. epidermidis* 275lp, satisfactory values of compound **1a** were achieved at 128 µg/mL (MIC_90_). A slightly lower activity, at 64 µg/mL (MIC_50_), was demonstrated for *S. aureus* RF 122, *E. faecalis* ATCC 29212, and *S. epidermidis* S22. In the case of *S. aureus* RF 122 at a concentration of 128 µg/mL, the eradication was high enough to establish MIC_90_. For *S. epidermidis* B145 and ATCC 12228, as well as *S. aureus* MRSAkj, MIC_50_ = 128 µg/mL was demonstrated. On the other hand, *S. epidermidis* achieved MIC_90_ for concentrations that were double that recorded for MRSA. Concentrations that were another two orders higher allowed MIC_90_ to be established for *S. epidermidis* B145. In contrast, the weakest effect of compound **1a** was shown against *S. epidermidis* ATCC12228, for which the MIC_50_ was >512 µg/mL.

A slightly weaker effect was shown for the sulfur derivative of (S, S)-(-)-camphor (**2a**). In contrast, the growth of *S. aureus* MRSAkj, *S. aureus* RF 122, *S. epidermidis* B145, and *S. epidermidis* 275lp was inhibited by 50% by this compound at a concentration of 128 µg/mL. In the case of *S. epidermidis* 275lp and *S. epidermidis* B145, the elimination was high enough at a concentration of 256 µg/mL to establish MIC_90_. The same MIC_90_ value was also determined for *S. epidermidis* ATCC12228 and *S. epidermidis* S22; however, at concentrations that were two ranges higher, a slightly higher MIC_50_ (256 µg/mL) was found for *S. epidermidis* ATCC 12228 and S22, while the highest (512 µg/mL) was observed for *S. aureus* ATCC 25923 as well as *E. faecalis* ATCC 29212 (>512 µg/mL).

A significantly weaker antimicrobial activity against Gram-positive strains was observed for the sulfur derivative of (R, R -(+)-camphor (**3a**). This compound inhibited, in the range of 50%, the growth of all tested strains of *S. epidermidis*, as well as two of the three tested strains of *S. aureus* (i.e., RF122, and MRSAkj) at the highest applied concentration of 512 µg/mL. For *S. epidermidis* 275lp, this value allowed MIC_90_ to be established (512 µg/mL). On the other hand, it remained inactive (MIC_50_ > 512 µg/mL) against *S. aureus* strain ATCC 25923 and *E. faecalis* ATCC29212.

The source compounds from which the sulfur derivatives were obtained showed significantly weaker activity. Compound **1** only inhibited the growth of *S. epidermidis* 275lp, with MIC_50_ and MIC_90_ values of 128 µg/mL, respectively. Furthermore, compound **2** showed negligible effects on the Gram-positive strains tested. Only one of the eight strains had an MIC_50_ of 512 µg/mL—*S. epidermidis* B145. On the other hand, compound **3** did not show antimicrobial activity against any of the tested Gram-positive strains.

It is interesting to note that the obtained sulfur derivatives proved to be weakly effective against the tested Gram-negative strains. Only **2a** showed activity against *P. aeruginosa* ATCC 27853 and only **1a** showed activity against *A. baumannii* ATCC 19606, obtaining an MIC_50_ value of 512 µg/mL.

The obtained MIC and MBC values for the tested strains are presented in Table 1.

Investigations of the minimum bactericidal concentrations of the tested sulfur derivatives of camphor showed their negligible activities. To our knowledge, we were the first to study the values of the MBCs for camphor derivatives which exhibit antibacterial properties. Bactericidal activity was observed only for **1a** and **2a**. Compound **1a** totally eradicated the tested *Staphylococcus* strains, i.e., MRSAkj and RF 122 (MBC values of 256 and 512 µg/mL, respectively), and 275lp or B145 (MBC = 256 µg/mL). On the other hand, compound **2a** showed a lethal effect against *S. epidermidis* B145 and *E. faecalis*, reaching MBC = 256 µg/mL, and a slightly weaker effect, at MBC = 512 µg/mL, against *S. aureus* (RF 122 and MRSAkj) and *S. epidermidis* 275lp. None of the analyzed compounds showed lethal activity against the tested Gram-negative strains.

Camphor is widely known for its antimicrobial activity [24,25,26,27,28,29] and many camphor derivatives have been studied for these purposes, often being used to synthesize very broad chemical compounds. Therefore, the overarching aim of our article was to demonstrate the effect of attaching a sulfur atom to a camphor molecule on increasing the compound’s antimicrobial and antibiofilm potential. Accordingly, we compared our results with data from the literature based on various sulfur derivatives of camphor. We did not focus on pure camphor, whose activity, as well as toxicity, is high. The changes we made to the original camphor molecule have shown great promise. So far, it has been reported that our modifications significantly improved camphor’s antimicrobial activity against both Gram-positive and Gram-negative strains of the modified compounds, e.g., semicarbazone vs. thiosemicarbazone [31,32], bezoxazole-2-thione vs. benzothiazole-2-thione [33], and uracil vs. 2-thiouracil [34]. Furthermore, insignificant antibacterial properties have been reported for dicamphor diselenide against both Gram-positive and Gram-negative pathogens. This compound did not show significant antimicrobial activity against most of the tested pathogens: the MIC was ≥250 µg/mL for *Pseudomonas aeruginosa* ATCC 15692, *S. aureus* ATCC 29213, and *S. epidermidis* ATCC 35984. These results are consistent with those obtained with our derivatives. Thus, linking two camphor molecules with a selenium bridge did not significantly increase their antimicrobial activity. Interestingly, a moderate activity of this compound was noted against *Streptococcus pyogenes* ATCC 20565 (MIC = 31.25 µg/mL), a strain not included in our study [35].

Mikláš et al. synthesized a group of homochiral quaternary ammonium sulfonamides, which are camphor derivatives. Several quaternary ammonium salts are known to exhibit strong antimicrobial activity and are widely used as disinfectants and antiseptics. Among the obtained compounds, the most active against *S. aureus* ATCC 6538 and *E. coli* CNCTC 377/79 was N-{2-[((1S,4R)-7,7-dimethyl-2-oxobicyclo[2.2.1]heptan-1-yl) methylsulfonamido]ethyl}-N,Ndimethyltetradecan-1-aminium bromide, which exhibited MIC values of 1.05 and 2.2 µmol/L, respectively [36].

The antibacterial activity of camphoryl pyrimidine amine derivatives was also evaluated, using microdilution assays, against clinical isolates of Gram-positive and Gram-negative bacteria. The derivative (N-(2,4-difluorobenzyl)-4-(4-methoxyphenyl)- 8,9,9-trimethyl-5,6,7,8-tetrahydro-5,8-methylquinazolin-2-amine) was particularly active, showing activity against *P. aeruginosa*, *E. coli*, and MRSA *S. aureus*, with MIC values of 16, 8, and 8 µg/mL, respectively. The camphor compound tested by the authors showed moderate activity against an *S. aureus* isolate (MIC = 16 µg/mL) [37]. It is worth noting that these compounds contain extended ring structures in their structure and can be considered structural analogs of the fungicide diflumetorim, also known for its potent antifungal activity. In addition, the activity of camphor-based thiazoles was tested against Gram-positive bacteria. The microdilution method evaluation showed MIC values for *S. aureus* and *S. epidermidis* of 0.98–7.81 µg/mL [38]. It is noteworthy that thiazoles are one of the most intensively studied classes of five-membered aromatic heterocycles and are known for their antimicrobial activity [39]. Weak antimicrobial activity against Gram-positive and Gram-negative strains was also observed for another thiazole derivative of camphor: 2-(2-((1S,4R,E)-1,7,7-trimethylbicyclo[2.2.1]heptane-2-ylidene) hydrazinyl)thiazol-5(4H)-one. The insertion of additional aromatic ring into this chemical compound did not result in a significant increase in its antimicrobial activity [40].

Carvalho’s team has published a series of articles on the antibacterial activity of camphor-silver derivative complexes [22,41,42]. The highest activities were shown by imine derivatives against *E. coli* ATCC 25922 (MIC value in the range of 37–59 µg/mL) and by sulfonylmine against *P. aeruginosa* 477 (MIC = 36 µg/mL). Much lower activity was observed against Gram-positive bacteria, i.e., the *S. aureus* strain. The Newman compounds that were the first group of compounds with a higher antimicrobial activity (MIC range: 47–151 µg/mL) than sulfonyl derivatives (MIC range: 114–257 µg/mL) [41].

A similar study also evaluated camphor compounds (imine, phenazine, sulfonimine) against Gram-positive and Gram-negative bacteria. The imine derivatives showed high activity against *E. coli* ATCC 25922 (MIC = 7.2–59.4 µg/mL) and *P. aeruginosa* 477 (MIC = 3.4–43 µg/mL) but lower activity against *S. aureus* Newman (MIC = 9.3–125 µg/mL). A comparable relationship was observed for the sulfoimine derivatives. These also showed higher activity against Gram-negative bacteria (for *E. coli*, MIC value was in the range of 15.5–125 µg/mL, while for *P. aeruginosa* it was between 6.7–125 µg/mL) and lower activity against Gram-positive bacteria (for *S. aureus*, the MIC ranged from 32.5 to 125 µg/mL). In contrast, the phenazine derivative was weakly active against *P. aeruginosa* (MIC = 68 µg/mL) and *E. coli* (MIC = 98 µg/mL), as well as *S. aureus* (MIC = 118 µg/mL) [22]. Other studies have also evaluated the antibacterial properties of Ag (I) complexes with camphor di-imines (mono-camphor, bi-camphor, camphor sulphonyl) against Gram-negative and Gram-positive bacteria. Significantly lower MIC values were shown for *P. aeruginosa* 477 (MIC values ranged from 19 to 138 µg/mL) and *E. coli* ATCC 25922 (MIC values: 20–123 µg/mL) than for *S. aureus* (MIC = 73–259 µg/mL). The lowest reported MIC values were presented by the silver complex with the bi-camphor derivative with the highest MIC values recorded by the derivative camphor sulphonylimine [42]. Peraman et al. evaluated the effects of aryl and heteryl derivatives of camphor (C) and camphor sulfonic acid (CSA) on Gram-negative and Gram-positive bacteria. In general, these camphor derivatives showed higher antimicrobial activity than the sulfonic derivatives. In the field of Gram-negative bacteria, the MIC values were as follows: *A. baumannii* ATCC 19606 8→64 µg/mL (C), 32→64 µg/mL (CSA), *P. aeruginosa* ATCC 27853 16–64 µg/mL (C), ≥64 µg/mL (CSA), and *E. coli* ATCC 25922: 16→64 µg/mL (C), 32→64 µg/mL (CSA). In the Gram-positive bacteria, a comparable pattern was observed with MRSA, where the MIC ranged from 8 to 64 µg/mL for C, and from 16 to 64 µg/mL for CSA [43].

### 2.2. Effect of Sulfur Derivatives of Camphor on Biofilm Formation

**Figure 2:**
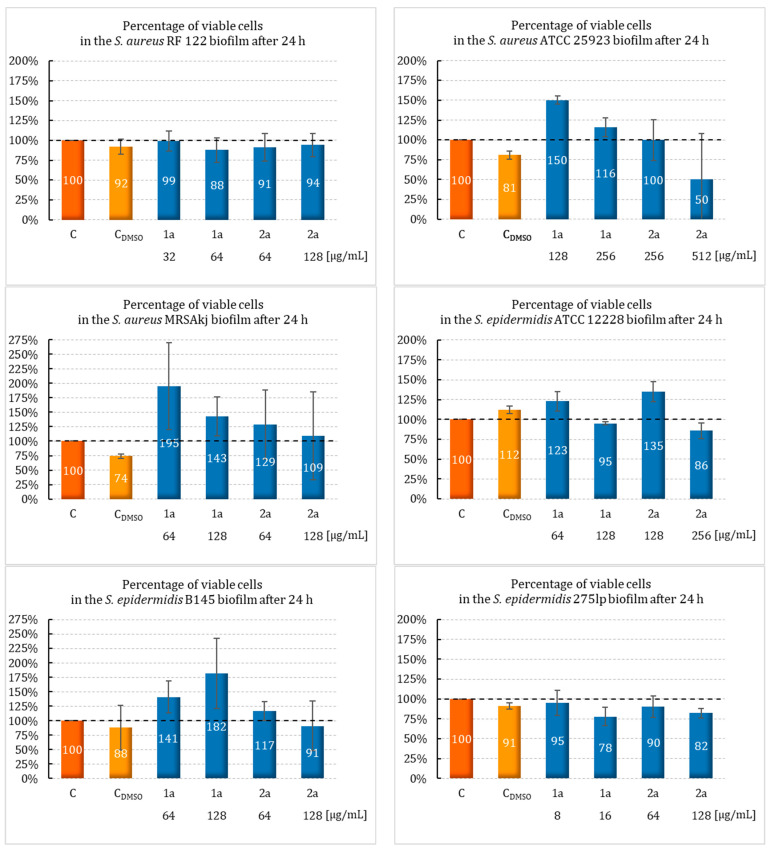

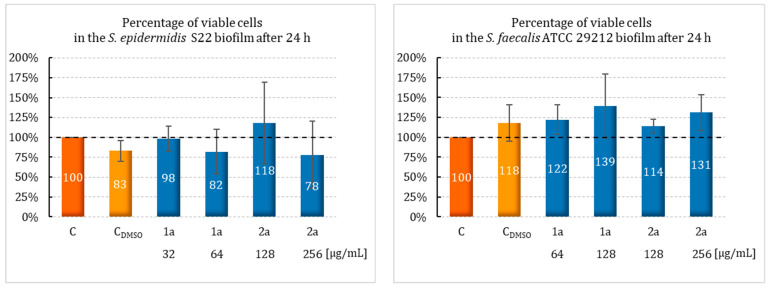
Impact of camphor derivatives **1a** and **2a** on bacterial biofilm formation. Legend: C (orange)—control (strain growth in TSB); C_DMSO_ (yellow)—effect of DMSO solvent on the growth of the tested strain.

The **1a** and **2a** compounds were selected for further study due to exhibiting the highest antimicrobial activity. To the best of our knowledge, this study was the first that evaluated the effects of sulfur camphor derivatives on the growth of bacterial biofilms in the presence of these derivatives and the eradication of mature biofilms after exposure to the newly synthesized camphor derivatives. To evaluate the biofilm-forming ability of *S. aureus*, *S. epidermidis*, and *E. faecalis*, these strains were incubated in the presence of sulfur derivatives of camphor. The biofilm was formed under stationary conditions within 24 h at MIC and ½ MIC concentrations. The biofilm formation by the bacteria was determined by TTC staining. The results are shown in Figure 2.

The most relevant information among the few available reports on the action of camphor compounds in inhibiting the growth of bacterial biofilms is provided by the study of Sancineto et al. [35]. Camphor diselenide was characterized by a strong ability to inhibit the growth of bacterial biofilms, especially Gram-positive strains. At the subinhibitory concentrations (0.2 MIC), camphor diselenide (6.25 µg/mL) showed excellent activity against *S. pyogenes* ATCC 20565 (% cell viability < 20%) and moderate activity (50 µg/mL) against *S. epidermidis* ATCC 35984 and *S. aureus* ATCC 29213 (viable cells decreased to ~70%). In the case of Gram-negative bacteria, there was no significant effect of this compound. Only *P. aeruginosa* ATCC 15692 showed the ability to reduce the cell viability to ~80% at a concentration of 50 µg/mL, while none of the Gram-negative strains tested by us were chosen for further investigation due to high MIC values. Our results indicate that attaching a single sulfur atom to the camphor chain yields outcomes comparable to those found in Sancineto et al.’s studies on dicamphor diselenide. However, it is important to remember the numerous studies indicating the toxicity of organoselenium compounds due to the generation of oxidative stress and its associated effects [44]. Our study did not show any significant effect of camphor sulfur derivatives on the biofilm formation process by the tested strains. Nevertheless, we observed that compound **2a** was more effective than compound **1a** in this regard. In addition, the strains *S. epidermidis* B145, *S. aureus* ATCC 25923, *S. aureus* MRSAkj, and *E. faecalis* showed a stimulating effect on the biofilm production in the presence of **1a**. In contrast, this compound has shown an effect on the planktonic forms produced by *S. epidermidis* 275lp, *S. epidermidis* S22, and *S. aureus* RF 122 (biofilm growth of 78%–99%). On the other hand, for *S epidermidis* ATCC 12228, in addition to probiofilm activity at ½ MIC (64 µg/mL), slight antibiofilm activity (5%) was observed at the MIC concentration (128 µg/mL).

A stronger antibiofilm effect occurred with compound **2a**. Although it was weak, it differed slightly from that shown by compound **1a**. The level of reduction of the planktonic forms was the highest for *S. epidermidis* S22. This strain showed a significant decrease, about 22% (*p* = 0.0008), in viable cells. Furthermore, this derivative was also shown to have a slight antibiofilm activity at MIC = 256 µg/mL (cell viability decreased to 86%) against *S. epidermidis* ATCC 12228). However, the lowest activity against biofilm formation was shown for *S. epidermidis* B145. Compound **2a**, at a concentration of 64 µg/mL (½ MIC), reduced the biofilm production capacity of this strain to 9%.

On the other hand, compound **2a**, at a concentration equal to the MIC (256 µg/mL), contributed to an increase in the reduction of planktonic forms in *S. aureus* ATCC 25925 by up to 50%. In addition, this compound showed moderate activity against *S. epidermidis* 275lp, contributing to an 18% and 10% reduction in the biofilm formation at MIC (128 µg/mL) and ½ MIC (64 µg/mL), respectively. A significantly weaker effect was observed for *S. aureus* RF 122; this derivative led to a 9% decrease in the biofilm formation at ½ MIC concentration (64 µg/mL).

For *S. aureus* MRSAkj and *E. faecalis* ATCC 29212, none of the evaluated compounds showed (at MIC and ½ MIC concentrations) the ability to inhibit biofilm formation in the presence of **2a**. Moreover, more than a 100% increase in cell viability was observed in both strains.

An interesting observation is the effect of the sulfur derivative (**2a**) on three strains of *S. epidermidis* (S22, ATCC 12228, and B145). Indeed, it was shown that a concentration of ½ MIC had a stimulating effect on the biofilm formation, in contrast to the MIC concentration, at which a reduction was observed.

### 2.3. Effect of Sulfur Derivatives of Camphor on Eradication of Mature Bacterial Biofilm

The ability to eradicate 24 h biofilms formed by the tested Gram-positive bacteria was evaluated for the **1a** and **2a** compounds. The growth of the biofilms was carried out under stationary conditions for 24 h at 37 °C. Concentrations of the sulfur derivatives of camphor, **1a** and **2a**, corresponding to the MIC, 2×MIC, and 4×MIC values were added to the created biofilms to determine their ability to eliminate previously formed biofilms. The tests were conducted with the use of sterile water for injection (H_2_Oinj) and TBS medium with glucose (TSBglu). The experiments were conducted in two extremely different environments due to our estimation of their effects on the compositions of the biofilm matrices, thus taking into account the differences due to species belonging. The eradicating effects of the derivatives on the formed biofilms were determined by TTC staining during 4 and 18 h incubation, respectively.

The results showed that both tested compounds, in the concentration range of MIC–4×MIC, are characterized by eradication activity against biofilms formed in H_2_Oinj. by *S. aureus*. It was also found that *S. aureus* MRSAkj can show an increase in its eradication effect in a concentration-dependent manner, depending on the derivative (eradication from MIC to 4×MIC in the range of 37–63% for **1a** and 37–58% for **2a**), and that *S. aureus* RF 122 showed stronger eradication at the MIC value than at the 4×MIC value (eradication at MIC/4×MIC for **1a** vs. **2a**—50/37% vs. 52/44%, respectively). On the other hand, for *S aureus* ATCC 25923, the highest reduction was shown at 57% for **2a** at a 2×MIC concentration and at 39% for **1a** at 2× and 4×MIC concentrations.

**Figure 3:**
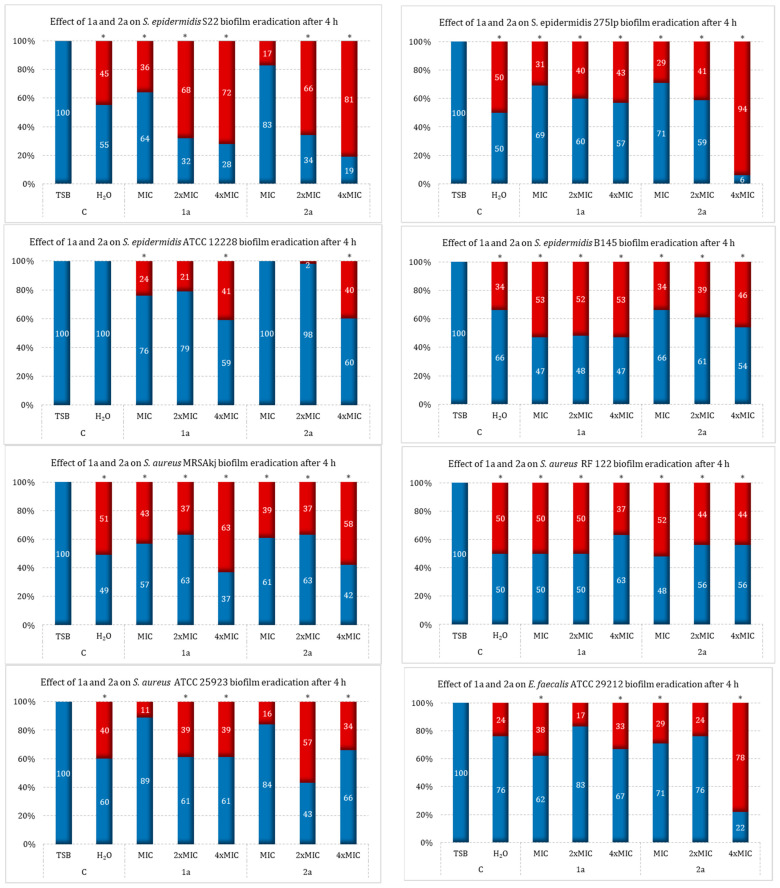
Impact of camphor derivatives (**1a** and **2a**) on bacterial biofilm eradication in sterile H_2_O medium; C—control (strain growth in TSB or H_2_O); blue—viability; red—mortality; *—*p* < 0.05. Legend: the numbers given in the OX axis signatures next to the compound number correspond to the ½×MIC and MIC values (expressed in µg/mL) for the strains tested: *S. aureus* RF 122 (for **1a**: ½×MIC = 32, MIC = 64; for **2a**: ½×MIC = 64, MIC = 128), *S. aureus* ATCC 25923 (for **1a**: ½×MIC = 128, MIC = 256; for **2a**: ½×MIC = 256, MIC = 512), *S. aureus* MRSAkj (for **1a**: ½×MIC = 64, MIC = 128; for **2a**: ½×MIC = 64, MIC = 128), *S. epidermidis* ATCC 12228 (for **1a**: ½×MIC = 64, MIC = 128; for **2a**: ½×MIC = 128, MIC = 256), *S. epidermidis* B145 (for **1a**: ½×MIC = 64, MIC = 128; for **2a**: ½×MIC = 64, MIC = 128), *S. epidermidis* 275lp (for **1a**: ½×MIC = 8, MIC = 16; for **2a**: ½×MIC = 64, MIC = 128), *S. epidermidis* S22 (for **1a**: ½×MIC = 32, MIC = 64; for **2a**: ½×MIC = 128, MIC = 256), *E. faecalis* ATCC 29212 (for **1a**: ½×MIC = 64, MIC = 128; for **2a**: ½×MIC = 128, MIC = 256).

The **1a** derivative was also shown to have a much weaker effect on *E. faecalis* ATCC 29212 biofilm (33% reduction) compared to **2a** (78% reduction). On the other hand, among the biofilms produced by the tested strains, *S. epidermidis* showed the lowest effect with ATCC 12228, which led to biofilm eradication at a level close to 40% with both **1a** and **2a** at a 4×MIC concentration. In contrast, the S22 strain showed a concentration-dependent reduction in the formed biofilm with both camphor derivatives: **1a** for MIC—4×MIC (in the range of 36–72%) and **2a** in a range that was almost two times higher, at 17–81%. A similar relationship was also observed for *S. epidermidis* 275lp biofilm. The **2a** derivative at a concentration of 4×MIC showed very strong antibiofilm activity—an eradication effect was observed at 94%. This compound at this concentration was the most active against this strain of all the preparations tested. The last of the tested strains, B145, was characterized by a moderate response to both camphor sulfur derivatives, with **1a** showing an eradication rate of about 50% over the full range of concentrations, while **2a** was active to a slightly weaker degree (eradication increased in proportion to concentration in a range of 34–46%). The results of the biofilm removal in H_2_Oinj. by the tested camphor sulfur derivatives are shown in Figure 3.

The divergence in eradication effectiveness may suggest that, for some strains, the antibiofilm effect is strictly dependent on the direct bactericidal activity of the camphor derivative. Nevertheless, it should be noted that, in the case of a well-formed biofilm, the penetration of a compound added at a high concentration may be difficult due to its limited availability to the bacterial cell (Figure 3) [45,46].

To examine the effect of the environment on the eradication capacity of the sulfur derivatives of the camphor derivatives **1a** and **2a**, this study was also carried out in TSBglu medium against the listed Gram-positive strains. Under these conditions, the **2a** derivative proved to be the most active. The most interesting results were obtained for the MRSAkj strain, with a cell viability reduction of 57% at 4×MIC. Similar effects of this compound were observed against *E. faecalis* (% eradication of viable cells at 56% for the highest concentration tested). A slightly weaker effect was obtained against *S. aureus* strain ATCC 25923 (47% cell viability reduction). Strains of *S. epidermidis* also exhibited the ability to eradicate biofilm at an interestingly high level. The highest activity was recorded for the **2a** derivative at a concentration of 4×MIC against strain B145 (eradication effect of 44%). In contrast, for the same derivative against strain S22, eradication was observed at a level that was not much lower, amounting to about 38% over the full range of concentrations. The lowest antimicrobial activity was observed for strain RF 122 (21% reduction at 4×MIC). In contrast, eradication for ATCC 12228 and 275lp was recorded only at the highest concentration and was ranked at 4% and 28%, respectively.

The **1a** derivative also proved to be the most active against the biofilm formed by the MRSAkj strain, showing an eradication of 68%. This compound had slightly weaker activity against *E. faecalis* ATCC 25923 and RF 122, for which an eradication effect of 34%, 33%, and 26%, respectively, was obtained at the 4×MIC concentration. Antibiofilm activity was not demonstrated (or was not significant) against the tested *S. epidermidis* strains, i.e., ATCC 12228 and 275lp (no activity), and S22 and B145 (% reduction of 3% and 8%, respectively). In addition, compound **2a** had higher activity than **1a** against the *E. faecalis* ATCC 29212 biofilm (eradication at all tested concentrations ranged from 27–56% for **2a** vs. 6–34% for **1a**).

**Figure 4:**
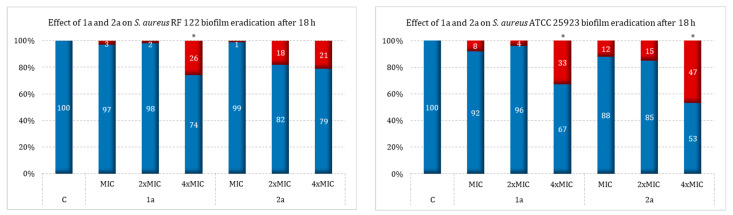

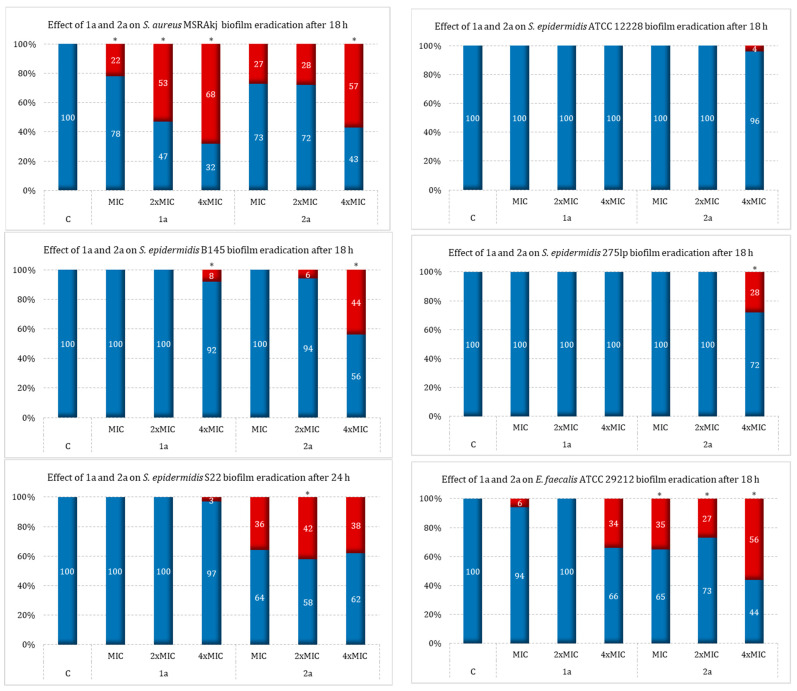
Impact of camphor derivatives (**1a** and **2a**) on bacterial biofilm eradication in TSBglu medium; C—control (strain growth in TSB); blue—viability; red—mortality; *—*p* < 0.05. Legend: the numbers given in the OX axis signatures next to the compound number correspond to the ½×MIC and MIC values (expressed in µg/mL) for the strains tested: *S. aureus* RF 122 (for **1a**: ½×MIC = 32, MIC = 64; for **2a**: ½×MIC = 64, MIC = 128), *S. aureus* ATCC 25923 (for **1a**: ½×MIC = 128, MIC = 256; for **2a**: ½×MIC = 256, MIC = 512), *S. aureus* MRSAkj (for **1a**: ½×MIC = 64, MIC = 128; for **2a**: ½×MIC = 64, MIC = 128), *S. epidermidis* ATCC 12228 (for **1a**: ½×MIC = 64, MIC = 128; for **2a**: ½×MIC = 128, MIC = 256), *S. epidermidis* B145 (for **1a**: ½×MIC = 64, MIC = 128; for **2a**: ½×MIC = 64, MIC = 128), *S. epidermidis* 275lp (for **1a**: ½×MIC = 8, MIC = 16; for **2a**: ½×MIC = 64, MIC = 128), *S. epidermidis* S22 (for **1a**: ½×MIC = 32, MIC = 64; for **2a**: ½×MIC = 128, MIC = 256), *E. faecalis* ATCC 29212 (for **1a**: ½×MIC = 64, MIC = 128; for **2a**: ½×MIC = 128, MIC = 256).

Two tested camphor derivatives showed moderate eradication activity against the *S. aureus* strains RF 122 and ATCC 25923. For the first one, the antimicrobial efficacy was 26% (**1a**), while for the second it was 47% (**2a**). However, these two activities were recorded at the highest concentrations, corresponding to 4×MIC. The results of this part of the study are shown in Figure 4.

Similar results to ours were reported in a study by Sancineto et al., where the effect of camphor diselenide on the eradication of a pre-formed biofilm of several Gram-positive pathogens was evaluated (among others, *S. pyogenes* ATCC20565, *S. aureus* ATCC29213, and *S. epidermidis* ATCC35984). The eradication ability was evaluated at substance concentrations of 0.1x, ½x, and MIC. The best results were obtained for *S. pyogenes* ATCC 20565, where the eradication was in the range of 30–40% (depending on the concentration). The results for *S. epidermidis* were similar and the eradication was higher at ½×MIC than at MIC, which may suggest that the inhibitory effect was not due to direct bactericidal activity (eradication <40%). In the case of *S. aureus* ATCC29213, the eradication was achieved up to a value of about 70% cell viability at a diselenide concentration equal to the MIC (for this strain, eradication proportional to the concentration of the substance was recorded). Importantly, the activity of the substance was lower in this case than for the previously mentioned Gram-positive strains [35].

An interesting relationship was observed in the analysis of our results. Both tested compounds showed comparable activity against the MRSAkj strain in both TSBglu and H_2_Oinj. cultures. Across the range of tested concentrations, a significantly stronger eradication was demonstrated for the **1a** derivative (range of antibiofilm activity: 32–68% vs. 37–63%; TSBglu vs. H_2_Oinj.) relative to **2a** (antibiofilm range: 27–54% vs. 37–58%; TSBglu vs. H_2_Oinj.). For the other strains, the environment had a significant effect on the level of biofilm eradication. Under more deficient conditions (H_2_Oinj.), the tested compounds showed higher antibiofilm activity than under enriched conditions (TSBglu).

### 2.4. Ability to Remove Biofilm under Flow Conditions

The tests conducted with the use of the Bioflux system showed that the sulfur derivatives of camphor (**1a** and **2a**) did not exhibit antimicrobial activity in the flow conditions (in contrast to stationary conditions) (Figure S11). To the best of our recent knowledge, there is no data in the literature on sulfur derivatives of camphor under flow-through conditions. Therefore, it is difficult to compare our results to results for other compounds.

## 3. Materials and Methods

### 3.1. Derivation of Sulfur Derivatives of Camphor

Column Chromatography and Purification: the reaction mixtures were purified using column chromatography with silica gel (Kieselgel 60, Merck, Darmstadt, Germany, mesh 230–400). The eluent (solvent) used was a mixture of hexane and diethyl ether in varying volume ratios.

Gas Chromatography (GC) Analysis: GC analyses were performed on an Agilent Technologies 6890 N gas chromatograph. The GC column used was an SGE BP5 GC column (5% phenyl/95% dimethyl polysiloxane, 30 m × 0.25 mm × 0.25 µm). Injector temperature: 150 °C, flame ionization detector (FID) temperature: 300 °C. Compounds were also analyzed using a gas chromatograph coupled to a mass spectrometer (Brucker 8900 GC-MS, 43X-GC, Brucker Daltonics, Billerica, MA, USA). Compound separation was carried out using a Zebron ZB-5 capillary column (30 m × 0.25 mm × 0.25 µm; Phenomenex, Torrance, CA, USA). GC-MS analysis parameters: scan range 35–320 *m*/*z*, helium (5.0) as the carrier gas (flow rate: 1.01 mL·min^−1^), split ratio: 1:30. Temperature program: from 50 °C (hold 2 min) to 160 °C at a rate of 2 °C·min^−1^, then to 300 °C (hold 6 min) at a rate of 20 °C for 5 min. Injection volume: 1 µL. For calculation of retention indices, according to *n*-alkane series, Excel macro [47] was applied. For comparison of RI and MS values, NIST23 database was used.

Other Techniques: NMR spectra were acquired using JEOL DeltaTM 400 MHz (JEOL USA, Inc., Peabody, MA, USA) and Bruker AvanceTM 600 MHz (Bruker, Rheinstetten, Germany) spectrometers (CDCl_3_ as the solvent). Optical rotation measurements of biotransformation products were conducted using a Jasco P-2000 polarimeter (Jasco, Easton, PA, USA) (solutions in chloroform, concentrations denoted in g/100 mL).

### 3.2. Synthesis to Obtain Thiocamphor

The synthesis was performed according to the well-known procedure [30]. The reaction of the racemic ketone as well as the pure enantiomers was conducted according to the same procedure. Briefly, 1.5 g of substrate (10 mmol), 8 g (20 mmol) of Lawesson’s reagent, and 30 mL of dry toluene were placed in a three-neck flask. The whole reaction was heated under a reflux condenser in a nitrogen atmosphere. The progress of the reaction was monitored by GC-FID. After 4 h, the reaction was stopped and cooled, and some of the solvent was evaporated. In the next step, the reaction mixture was dissolved in a hexane/Et_2_O mixture (*v*/*v*, 10:1) and adsorbed on silica gel, and then the crude product was purified on a column using hexane:diethyl ether eluent (*v*/*v*, 10:1).

The efficiencies, and physical and chemical constants, of the obtained sulfur derivatives are given below:

(±)-1,7,7-trimethylbicyclo[2.2.1]heptane-2-thione (**1a**): 1.13 g (yield 67%), KI = 1248 (estimated semistandard non-polar retention index according base NIST23 KI = 1258, AI predicted 1298); ^1^H NMR (600 MHz, CDCl_3_): 2.76 (dm, J= 20.8 Hz, ^1^H, one of CH_2_-3), 2.39 (d, J= 20.8 Hz, ^1^H, one of CH_2_-3), 2.15 (dd, J =4.8, 4.4 Hz, ^1^H, CH-4), 1.97 (m, ^1^H, one of CH_2_-5), 1.74 (m, ^1^H, one of CH_2_-6), 1.30 (m, 2H, one of CH_2_-5 and one of CH_2_-6), 1.08 (s, 3H, CH_3_), 1.02 (s, 3H, CH_3_), 0.77 (s, 3H, CH_3_); ^13^C NMR 271.9 (C-2), 69.3 (C-1), 55.6 (C-3), 49.0 (C-7), 45.2 (C-4), 33.9 (C-6), 27.3 (C-5), 19.9 (C-8), 19.7 (C-9), 13.2 (C-10)

(1S,4S)-(+)-1,7,7-trimethylbicyclo[2.2.1]heptane-2-thione (**2a**): 0.79 g (yield 47%), ${\left[a\right]}_{D}^{23}$ = +36.8 (c = 0.37, CHCl_3_)

(1R,4R)-(-)-1,7,7-trimethylbicyclo[2.2.1]heptane-2-thione (**3a**): 0.95 g, (yield 57%), ${\left[a\right]}_{D}^{23}$ = −39.2 (c = 0.41, CHCl_3_)

### 3.3. Tested Bacterial Strains

The planned study was performed on 16 bacterial strains: 8 Gram-positive (4 belonged to clinical strains *S. aureus* MRSAkj, *S. epidermidis* B145 (MRCNS-meticillin-resistant coagulase negative streptococci), *S. epidermidis* 275lp (MRCNS), and *S. epidermidis* S22 (MRCNS), and 4 were reference strains: *S. aureus* RF 122, *S. aureus* ATCC 25923, *S. epidermidis* ATCC 12228, and *E. faecalis* ATCC 29212) and 8 Gram-negative (both clinical: *E. coli* 1471 (ESBL+—extended-spectrum beta-lactamases), *E. coli* 27/2021, *E. coli* 105/2021, *E. coli* PA 170 (ESBL+), and reference: *E. coli* PCM 2427, *E coli* ATCC 35218, *A. baumannii* ATCC 19606, *P. aeruginosa* ATCC 27853) bacteria. All of the above-mentioned strains were obtained from museum collections located in the Department of Microbiology at the Wroclaw Medical University and the Department of Food Hygiene and Consumer Health Protection at the Wroclaw University of Life Sciences.

The resistance profiles of the “wild” strains taken for the experiments presented in this article are given in Table 2.

The deep-frozen strains were revived in tryptic-soy broth (TSB) for 24 h incubation at 37 °C under shaking conditions (125 rpm, rounds per minute) and isolated onto dedicated agar.

### 3.4. Assay of the Minimum Inhibitory and Bactericidal Concentrations

The minimum inhibitory concentrations (MICs) of the tested compounds were determined by the microdilution method on a microtiter plate based on the standards specified by the European Committee on Antimicrobial Susceptibility Testing (EUCAST) [48]. The experiment was performed using 18–20 h bacterial cultures on tryptic-soy agar (TSA) incubated at 37 °C. The baseline density of the bacterial strains tested was set to 0.5 MF (McFarland unit), 10^8^ CFU/mL in Muller–Hinton broth (MHB). The bacterial cultures used in the experiment were diluted to obtain a final density of 5 × 10^5^ CFU/mL in the well of the microtiter plate. The tested compounds, (±)-camphor (**1**), sulfur derivative (±)-camphor (**1a**), (S, S)-(−)-camphor (**2**), (S, S)-(+)-thiocamphor (**2a**), (R, R)-(+)-camphor (**3**), and (R, R)-(−)-thiocamphor) (**3a**), were diluted in DMSO, obtaining starting concentrations of 20 mg/mL. Subsequently, a series of dilutions of the tested sulfur derivatives of camphor were made in MHB medium in the concentration range of 1024 µg/mL–16 µg/mL. The tested bacterial strain was added to each well with the appropriate concentration, obtaining final dilution of the compounds of 512–8 µg/mL. Furthermore, the following controls were set for each assay: blank (pure MHB medium), growth (tested bacterial strain in MHB), background of tested compounds (compounds in the range of tested concentrations in MHB), and solvent (DMSO in the range of tested concentrations in MHB). Microtiter plates were incubated at 37 °C for 18 h (for *S. aureus*, *S. epidermidis*, *E. coli*, *P. aeruginosa*, and *A. baumannii* strains) or 24 h (for *E. faecalis*), followed by an initial visual MIC reading and then an automated reading at OD600 on a microplate reader (ASYS UMV340 Biochrom. Cambridge, UK). Determinations were performed using three experiments, each in three independent replicates.

The MIC_50_ values, expressed as % of viable bacteria (50% of bacteria that survived in the presence of the defined concentrations of the tested compounds), were calculated based on the average OD600 obtained from 3 replicates in each experiment relative to the growth control of the tested strain, amounting to 100%. The background of the compound and the blank control were included in the calculations, and were subtracted from the background of camphor sulfur derivatives at the tested concentrations and the growth control, respectively.

In order to determine the minimum bactericidal concentration (MBC), 5 µL each of well with MIC, as well as 1 well previous to it and 2 wells following it, was seeded on the TSA sector. Plates were incubated for 24 h at 37 °C, after which the growth of the tested bacterial strains was read visually by counting the grown bacterial colonies. Inoculation was performed in triplicate for each well.

### 3.5. Sulfur Derivatives’ Effect on Biofilm Formation

The biofilm-forming ability of the tested compounds was examined using a modified procedure described previously [49]. After an overnight cultivation (18–20 h), several colonies of the tested bacterial strains were transferred from TSA agar to 20 mL of TSB medium with 0.25% glucose (TSBglu), and then incubated for another 18–20 h at 37 °C with continuous shaking (125 rpm). In the next step, the culture was washed twice with 0.9% NaCl solution, and centrifuged each time at 4.500 rpm for 15 min. The resulting bacterial precipitate was resuspended in pure TSBglu medium and its optical density was determined as 0.5 MF, which corresponded to 10^8^ CFU/mL. In a further step, the bacterial suspension was diluted 100-fold in TSBglu. A series of dilutions of 2-fold-concentrated sulfur derivatives of camphor, **1a** and **2a**, were prepared, 256–16 µg/mL and 1024–128 µg/mL, respectively. The previously prepared bacterial cultures were then added to the wells of the titration plate, while diluting the cultures to 5 × 10^5^ CFU/mL and the compound solutions to 512–8 µg/mL. For each assay, a solvent control (DMSO in TSBglu), a growth control (the bacterial strain under study in TSBglu), and a blank control (pure TSBglu medium) were supplied. The plates were incubated for 24 h at 37 °C under steady-state conditions. At the end of the incubation, the fluid from the wells was collected and the plates were washed 2 times with 0.9% NaCl. The plate was thus prepared: 100 µL of TSBglu with 2,3,5-triphenyltetrazolium chloride (TTC) was added at a final concentration of 0.02% (starting concentration of 1%) per well. The plate was then incubated for 2 or 3 h for *Staphylococcus* spp. and *Enterococcus* spp., respectively, at 37 °C under steady-state conditions in the darkroom. After the completed incubation, the wells were rinsed again and then gently dried. A total of 100 µL of methanol:acetone mixture (*v*/*v*, 4:1) was added and shaken intensively at 350 rpm at RT for 15 min. After this time, absorbance (OD = 500 nm) was measured in a microplate reader (ASYS UMV340 Biochrom. Cambridge, UK). The ability to form biofilm in the presence of the tested compounds was evaluated in at last two experiments, each in three independent replicates.

### 3.6. Effect of Sulfur Derivatives of Camphor on Bacterial Biofilm Eradication

The ability of sulfur derivatives of camphor to eradicate the biofilm formed by the tested strains was performed based on the previously described procedure with modifications [50]. The experiment was conducted on overnight (18–20 h) cultures of the tested strains in TSBglu medium at 37 °C cultured under shaking conditions (125 rpm). The bacterial precipitate obtained after 2-fold centrifugation and washing with 0.9% NaCl was resuspended in pure TSBglu and an optical density of 0.5 MF (10^8^ CFU/mL) was determined. The pre-prepared bacterial cultures were diluted 30-fold in TSBglu and transferred to the wells of a 96-well microtiter plate. Incubation was carried out for 24 h at 37 °C under stationary conditions to produce a bacterial biofilm.

After that, the medium was collected from the wells and washed 2 times with 0.9% NaCl solution. To the so-prepared wells were added 100 µL of the tested compounds at concentrations of 4×MIC, 2×MIC, and MIC suspended in sterile water for injection (H_2_Oinj.) or in TSBglu. Incubation was performed at 37 °C under stationary conditions for 4 h or 18 h for compounds in water and TSBglu, respectively. After this time, the compounds were collected, the wells were washed, and TTC was added at a final concentration of 0.02% (starting concentration of 1%) in TSBglu. After 2 h (for *Staphylococcus* spp.) or 3 h (for *Enterococcus* spp.) incubation of the microtiter plate at 37 °C under stationary conditions in the dark, the wells were rinsed again, gently dried, and treated with a solution of methanol:acetone (*v*/*v*, 4:1). After intensively shaking (350 rpm at RT) for 15 min, absorbance was measured at OD = 500 nm in a microplate reader (ASYS UMV340 Biochrom. Cambridge, UK). The ability of biofilm eradication in the presence of the tested compounds was evaluated in four replicates in at least four experiments.

### 3.7. Influence of Flow Conditions on Biofilm Eradication

To determine the effect of dynamic flow of the culture medium on the biofilm eradication ability of the tested sulfur derivatives of camphor, Bioflux 1000z system (Fluxion, San Francisco, CA, USA) with a coupled environmental chamber microscope (Pecton Incubator XLS1, Carl Zeiss, Jena, Germany) was used. The experiment was conducted on a sulfur derivative of camphor in the (S)-(+) conformation, as it showed the most promising results in biofilm eradication under stationary conditions. Before the respective experiment, all channels of specialized microfluidic plates (Fluxion, San Francisco, CA, USA) were washed with 100 µL of TSBglu medium heated to 37 °C. A high intensity flow of 10 dynes/cm^2^ for 10 s was applied.

An 18–24 h liquid bacterial culture obtained in a TSB under shaking conditions at 125 rpm with a fixed density of 0.5 on the MF scale, corresponding to 10^8^ CFU/mL, was used for the experiment.

The so-prepared cultures of the tested bacterial strains (1 mL) were gently added to the entry channel (input). Preliminarily, a fast flow rate of 5 dynes/ cm^2^ for 1 s was released, and then changed to 0.1 dynes/cm^2^ for 24 h to enable the bacteria to successfully form a biofilm on the surface of the microcapillaries. After the incubation time, both the input and output wells were emptied of culture debris and gently rinsed with 0.9% NaCl solution. Then, 0.5 mL of H_2_Oinj. and a 4×MIC solution of **2a** prepared in H_2_Oinj were added to the input well for the negative control (Neg) and test sample (ST), respectively. Biofilm eradication capacity was carried out for 60 min at a slow flow rate of 0.1 dynes/cm^2^.

Following incubation, the area occupied by bacterial cells adhering to the surface of microcapillaries (Neg) relative to **2a** treated cells (ST) was determined. Images from different sections of treated microcapillaries were evaluated every 1 min for 60 min. The study was conducted in 3 independent experiments, each in 3 technical replicates.

### 3.8. Statistical Analysis

The experiments were performed in at least three independent replicates. Using Python’s SciPy package, mean values, standard deviations, and statistical significance were computed for each concentration of the tested compounds both for their biofilm formation and in eradication assays. Welch’s one-tailed *t*-test was applied to assess whether each compound significantly reduced absorbance compared to the growth control, with significance set at *p* < 0.05.

## 4. Conclusions

The exploration of natural compounds to support medicine is an ongoing topic of interest for researchers around the world. In our research, we examined in more detail the antimicrobial activity of sulfur derivatives of camphor. On the basis of our experiments, we can conclude that the studied compounds are not suitable for bacterial control in infusion form, but they perform excellently in the eradication of biofilm formed by strains under stationary conditions. The results we obtained make an important contribution to this field of science. Our experiments show that attaching a sulfur atom in the (S,S)-(+) conformation to camphor significantly improves both its antimicrobial and antibiofilm properties against Gram-positive strains. These results are very promising in the context of replacing camphor added to cosmetics with this compound. However, the experimental field needs to be greatly expanded to obtain a clear answer to the question of its possible use as a preventive agent in infections. Future studies directed at the likelihood of using compound **2a** as an additive to antiseptics and disinfectants would also be interesting. However, the initiation of such a procedure requires many years of scientific research involving, among other things, experiments first on eukaryotic cells and then on animals.

Furthermore, it is interesting to note that the best biofilm eradication results were obtained for the zoonotic strain *S. epidermidis* 275lp, which was isolated from a cow’s udder. Therefore, it is worth considering the possibility of using camphor sulfur derivatives in the treatment of skin infections in cattle.

## Figures and Tables

**Table 1:** MIC and MCB values of camphor derivatives against testes bacterial strains (µg/mL).

Bacterial Strain	MIC_50_/MIC_90_/MBC Values for Camphor Sulfur Derivatives (µg/mL)					
	1	2	3	1a	2a	3a
*S. aureus* RF 122	>512/>512/>512	>512/>512/>512	>512/>512/>512	64/128/128	128/>512/>512	512/>512/>512
*S. aureus* ATCC 25923	>512/>512/>512	>512/>512/>512	>512/>512/>512	256/>512/>512	512/512/>512	>512/>512/>512
*S. aureus* MRSAkj	>512/>512/>512	>512/>512>512	>512/>512>512	128/256/>512	128/>512>512	512/>512/>512
*S. epidermidis* ATCC 12228	>512/>512/>512	>512/>512/>512	>512/>512/>512	>512/>512/>512	256/512/>512	512/>512/>512
*S. epidermidis* 275lp	128/128/256	>512/>512/>512	>512/>512/>512	16/256/256	128/256/>512	512/512/>512
*S. epidermidis* B145	>512/>512/>512	512/>512/>512	>512/>512/>512	128/>512/>512	128/256/>512	512/>512/>512
*S. epidermidis* S22	>512/>512/>512	>512/>512/>512	>512/>512/>512	64/64/128	256/512/>512	512/>512/>512
*E. faecalis* ATCC 29212	>512/>512/>512	>512/>512/>512	>512/>512/>512	64/128/128	>512/>512/>512	>512/>512/>512
*E. coli* PCM 2427	>512/>512/>512	>512/>512/>512	>512/>512/>512	>512/>512/>512	512/>512/>512	>512/>512/>512
*E. coli * ATCC 35218	>512/>512/>512	>512/>512/>512	>512/>512/>512	>512/>512/>512	>512/>512/>512	>512/>512/>512
*E. coli* 1471	>512/>512/>512	>512/>512/>512	>512/>512/>512	>512/>512/>512	>512/>512/>512	>512/>512/>512
*E. coli* PA170	>512/>512/>512	>512/>512/>512	>512/>512/>512	>512/>512/>512	>512/>512/>512	>512/>512/>512
*E. coli* 27/2021	>512/>512/>512	>512/>512/>512	>512/>512/>512	>512/>512/>512	>512/>512/>512	>512/>512/>512
*E. coli* 105/2021	>512/>512/>512	>512/>512/>512	>512/>512/>512	>512/>512/>512	>512/>512/>512	>512/>512/>512
*A. baumannii* ATCC 19606	>512/>512/>512	>512/>512/>512	>512/>512/>512	512/>512/>512	>512/>512/>512	>512/>512/>512
*P. aeruginosa* ATCC 27853	>512/>512/>512	>512/>512/>512	>512/>512/>512	>512/>512/>512	512/>512/>512	>512/>512/>512

**Table 2:** Antimicrobial profiles determined by disc diffusion method.

Bacterial Strain	Antimicrobial Resistance Profile
*S. aureus* MRSAkj	FOX^R^, E^R^, CLD^R^, CIP^R^, GM^S^, TET^S^, SXT^S^, LZD^S^
*S. epidermidis* 275lp	FOX^S^, E^S^, CLD^S^, GM^S^, TET^S^, CIP^S^, SXT^S^, LZD^S^
*S. epidermidis* B145	FOX^R^, E^R^, CLD^R^, GM^R^, TET^R^, CIP^WZE^, SXT^S^, LZD^S^, VA^S^, TEC^S^
*S. epidermidis* S22	FOX^R^, E^R^, CLD^R^, SXT^R^, CIP^S^, VAN^S^, GM^S^, NET^S^, TEC^S^, TET^S^
*E. coli* 1471	CIP^R^, GM^R^, TOB^R^, CTX^R^, CAZ^R^, CXM^R^, AMC^R^, ATM^R^, FEP^R^, TZP^R^, MEM^R^, AKN^R^, SAM^R^, SXT^S^, IMP^S^, ETP^S^, DOR^S^
*E. coli* PA 170	CXM^R^, FEP^R^, CTX^R^, CAZ^R^, GM^R^, AMX^R^, SAM^R^, AMC^R^, TIM^R^, ATM^R^, TOB^R^, AKN^R^, CIP^R^, SMX^R^, SXT^R^, IMP^S^, MEM^S^
*E. coli* 27/2021	CXM^R^, FEP^S^, CAZ^S^, GM^S^, AMX^S^, SAM^S^, AMC^S^, TIM^S^, ATM^S^, TOB^S^, AKN^S^, CIP^S^, SXT^S^, IMP^S^, MEM^S^
*E. coli* 105/2021	CXM^R^, FEP^S^, CAZ^S^, GM^S^, AMX^S^, SAM^S^, AMC^S^, TIM^S^, ATM^S^, TOB^S^, AKN^S^, CIP^S^, SXT^S^, IMP^S^, MEM^S^

AKN (amikacin), AMC (amoxicillin/clavulanic acid), AMX (amoxicillin), ATM (aztreonam), CAZ (ceftazidime), CIP (ciprofloxacin), CLD (clindamycin), CTX (cefotaxime), CXM (cefuroxime), DOR (doripenem), E (erythromycin), ETP (ertapenem), FEP (cefepime), FOX (cefoxitin), GM (gentamicin), IMP (imipenem), LZD (linezolid), MEM (meropenem), NET (netilmicin), R (resistant), S (sensitive), SAM (ampicillin/sulbactam), SMX (sulfamethoxazole), SXT (co-trimoxazole), TET (tetracycline), TEC (teicoplanin), TIM (ticarcillin/clavulanic acid), TOB (tobramycin), TZP (piperacillin/tazobactam), VA (vancomycin), WZE (susceptible increased exposure).

## Data Availability

The data presented in this study are available in Supplementary Materials and upon request from the corresponding author.

## Supplementary Materials

The following supporting information can be downloaded at: https://www.mdpi.com/article/10.3390/ijms252010895/s1.

## References

[1] Yue H., Umehara Y., Trujillo-Paez J.V., Peng G., Nguyen H.L.T., Chieosilapatham P., Kiatsurayanon C., Song P., Okumura K., Ogawa H. (2021). Exogenous Factors in the Pathogenesis of Atopic Dermatitis: Irritants and Cutaneous Infections. Clin. Exp. Allergy.

[2] Gilaberte Y., Prieto-Torres L., Pastushenko I., Juarranz Á. (2016). Anatomy and Function of the Skin.

[3] Bouza E., De Rosa F.G., Guzek A., Dirschka T., Pellacani G. (2022). Multidisciplinary Panel Opinion on the Management of Bacterial Skin Infections. JEADV Clin. Pract..

[4] Byrd A.L., Belkaid Y., Segre J.A. (2018). The Human Skin Microbiome. Nat. Rev. Microbiol..

[5] Lee H.J., Kim M. (2022). Skin Barrier Function and the Microbiome. Int. J. Mol. Sci..

[6] Skowron K., Bauza-kaszewska J., Kraszewska Z., Wiktorczyk-kapischke N., Grudlewska-buda K., Kwiecińska-piróg J., Wałecka-zacharska E., Radtke L., Gospodarek-komkowska E. (2021). Human Skin Microbiome: Impact of Intrinsic and Extrinsic Factors on Skin Microbiota. Microorganisms.

[7] Nowicka D., Chilicka K., Dzieńdziora-Urbińska I. (2022). Host-Microbe Interaction on the Skin and Its Role in the Pathogenesis and Treatment of Atopic Dermatitis. Pathogens.

[8] Ki V., Rotstein C. (2008). Bacterial Skin and Soft Tissue Infections in Adults: A Review of Their Epidemiology, Pathogenesis, Diagnosis, Treatment and Site of Care. Can. J. Infect. Dis. Med. Microbiol..

[9] Golan Y. (2019). Current Treatment Options for Acute Skin and Skin-Structure Infections. Clin. Infect. Dis..

[10] Dryden M.S. (2010). Complicated Skin and Soft Tissue Infection. J. Antimicrob. Chemother..

[11] Negut I., Grumezescu V., Grumezescu A.M. (2018). Treatment Strategies for Infected Wounds. Molecules.

[12] Holder I.P., Campa M., Bendinelli M., Friedman H. (1993). Aeruginosa Burn Infections: Pathogenesis and Treatment. Pseudomonas aeruginosa as an Opportunistic Pathogen. Infectious Agents and Pathogenesis.

[13] Hofstee M.I., Muthukrishnan G., Atkins G.J., Riool M., Thompson K., Morgenstern M., Stoddart M.J., Richards R.G., Zaat S.A.J., Moriarty T.F. (2020). Current Concepts of Osteomyelitis: From Pathologic Mechanisms to Advanced Research Methods. Am. J. Pathol..

[14] Qin S., Xiao W., Zhou C., Pu Q., Deng X., Lan L., Liang H., Song X., Wu M. (2022). *Pseudomonas aeruginosa*: Pathogenesis, Virulence Factors, Antibiotic Resistance, Interaction with Host, Technology Advances and Emerging Therapeutics. Signal Transduct. Target. Ther..

[15] Cavallo I., Sivori F., Mastrofrancesco A., Abril E., Pontone M., Di Domenico E.G., Pimpinelli F. (2024). Bacterial Biofilm in Chronic Wounds and Possible Therapeutic Approaches. Biology.

[16] Li Z., Xu X., Leng X., He M., Wang J., Cheng S., Wu H. (2017). Roles of Reactive Oxygen Species in Cell Signaling Pathways and Immune Responses to Viral Infections. Arch. Virol..

[17] Zegadło K., Gieroń M., Żarnowiec P., Durlik-Popińska K., Kręcisz B., Kaca W., Czerwonka G. (2023). Bacterial Motility and Its Role in Skin and Wound Infections. Int. J. Mol. Sci..

[18] Xiao S., Yu H., Xie Y., Guo Y., Fan J., Yao W. (2021). The Anti-Inflammatory Potential of *Cinnamomum camphora* (L.) J.Presl Essential Oil in Vitro and in Vivo. J. Ethnopharmacol..

[19] Sounouvou H.T., Toukourou H., Catteau L., Toukourou F., Evrard B., Van Bambeke F., Gbaguidi F., Quetin-Leclercq J. (2021). Antimicrobial Potentials of Essential Oils Extracted from West African Aromatic Plants on Common Skin Infections. Sci. African.

[20] Orchard A., Van Vuuren S. (2017). Commercial Essential Oils as Potential Antimicrobials to Treat Skin Diseases. Evid.-Based Complement. Altern. Med..

[21] Wang W., Li D., Huang X., Yang H., Qiu Z., Zou L., Liang Q., Shi Y., Wu Y., Wu S. (2019). Study on Antibacterial and Quorum-Sensing Inhibition Activities of Cinnamomum camphora Leaf Essential Oil. Molecules.

[22] Costa J., Sousa S., Galvão A., Mata J., Leitão J., Carvalho M. (2021). Key Parameters on the Antibacterial Activity of Silver Camphor Complexes. Antibiotics.

[23] Santos T.B., Vieira A.A., Paula L.O., Santos E.D., Radi P.A., Khouri S., Maciel H.S., Pessoa R.S., Vieira L. (2017). Flexible Camphor Diamond-like Carbon Coating on Polyurethane to Prevent Candida Albicans Biofilm Growth. J. Mech. Behav. Biomed. Mater..

[24] Duda-Madej A., Viscardi S., Grabarczyk M., Topola E., Kozłowska J., Mączka W., Wińska K. (2024). Is Camphor the Future in Supporting Therapy for Skin Infections?. Pharmaceuticals.

[25] Chen W., Vermaak I., Viljoen A. (2013). Camphor—A Fumigant during the Black Death and a Coveted Fragrant Wood in Ancient Egypt and Babylon—A Review. Molecules.

[26] Ware R.A., Ghude D.S., Akolkar M.P., Bhise M.G. (2020). The Therapeutic and Medicinal Use of Camphor in Ordinary Life. Int. J. Res. Eng. Sci. Manag..

[27] Zielińska-Błajet M., Feder-Kubis J. (2020). Monoterpenes and Their Derivatives—Recent Development in Biological and Medical Applications. Int. J. Mol. Sci..

[28] Zuccarini P., Soldani G. (2009). Camphor: Benefits and Risks of a Widely Used Natural Product Pharmacology Pharmacodynamics. Acta Biol. Szeged..

[29] Malabadi R.B., Kolkar K.P., Meti N.T., Chalannavar R.K. (2021). An age old botanical weapon for herbal therapy: Camphor tree, *Cinnamomum camphora*. Int. J. Innov. Sci. Res. Rev..

[30] Shimada K., Kodaki K., Aoyagi S., Takikawa Y., Kabuto C. (1999). Formation of Kinetically Stabilized Dithiiranes by Treating Thione S-Oxides Bearing a Bulky Substituent with Lawesson’s Reagent. Chem. Lett..

[31] Raghav N., Kaur R. (2014). Synthesis and Evaluation of Some Semicarbazone- and Thiosemicarbazone-Based Cathepsin B Inhibitors. Med. Chem. Res..

[32] Olanrewaju A.A., Oke D.G., Adekunle D.O., Adeleke O.A., Akinola O.T., Emmanuel A.V., Oyeneyin O.E. (2023). Synthesis, in-Vitro and in-Silico Antibacterial and Computational Studies of Selected Thiosemicarbazone-Benzaldehyde Derivatives as Potential Antibiotics. SN Appl. Sci..

[33] Jasiewicz B., Babijczuk K., Warżajtis B., Rychlewska U., Starzyk J., Cofta G., Mrówczyńska L. (2023). Indole Derivatives Bearing Imidazole, Benzothiazole-2-Thione or Benzoxazole-2-Thione Moieties—Synthesis, Structure and Evaluation of Their Cytoprotective, Antioxidant, Antibacterial and Fungicidal Activities. Molecules.

[34] Marinova P., Tamahkyarova K. (2024). Synthesis and Biological Activities of Some Metal Complexes of Peptides: A Review. BioTech.

[35] Sancineto L., Piccioni M., De Marco S., Pagiotti R., Nascimento V., Braga A.L., Santi C., Pietrella D. (2016). Diphenyl Diselenide Derivatives Inhibit Microbial Biofilm Formation Involved in Wound Infection. BMC Microbiol..

[36] Mikláš R., Miklášová N., Bukovský M., Horváth B., Kubincová J., Devínsky F. (2014). Synthesis, Surface and Antimicrobial Properties of Some Quaternary Ammonium Homochiral Camphor Sulfonamides. Eur. J. Pharm. Sci..

[37] Zhang H., Huang T., Liao X., Zhou Y., Chen S., Chen J., Xiong W. (2022). Extraction of Camphor Tree Essential Oil by Steam Distillation and Supercritical CO_2_ Extraction. Molecules.

[38] Laczkowski K., Misiura K., Biernasiuk A., Malm A., Siwek A., Plech T., Ciok-Pater E., Skowron K., Gospodarek E. (2015). Synthesis, In Vitro Biological Screening and Molecular Docking Studies of Novel Camphor-Based Thiazoles. Med. Chem..

[39] Mohanty P., Behera S., Behura R., Shubhadarshinee L., Mohapatra P., Barick A.K., Jali B.R. (2022). Antibacterial Activity of Thiazole and Its Derivatives: A Review. Biointerface Res. Appl. Chem..

[40] Naghiyev F.N., Asgarova A.R., Maharramov A.M., Rahimova A.G., Akhundova M.A., Mamedov I.G. (2020). Synthesis and Antimicrobial Properties of Some Thiazole and Pyridine Derivatives. New Mater. Compd. Appl..

[41] Carvalho M.F.N.N., Leite S., Costa J.P., Galvão A.M., Leitão J.H. (2019). Ag(I) Camphor Complexes: Antimicrobial Activity by Design. J. Inorg. Biochem..

[42] Cardoso J.M.S., Galvão A.M., Guerreiro S.I., Leitão J.H., Suarez A.C., Carvalho M.F.N.N. (2016). Antibacterial Activity of Silver Camphorimine Coordination Polymers. Dalt. Trans..

[43] Peraman R., Tiwari A.K., Geetha Vani M., Hemanth J., Geetha Sree Y., Karthik K., Ashby C.R., Padmanabha Reddy Y., Pemmidi R.V. (2018). New Camphor Hybrids: Lipophilic Enhancement Improves Antimicrobial Efficacy against Drug-Resistant Pathogenic Microbes and Intestinal Worms. Med. Chem. Res..

[44] Nogueira C.W., Barbosa N.V., Rocha J.B.T. (2021). Toxicology and Pharmacology of Synthetic Organoselenium Compounds: An Update. Arch. Toxicol..

[45] Lebeaux D., Ghigo J.-M., Beloin C. (2014). Biofilm-Related Infections: Bridging the Gap between Clinical Management and Fundamental Aspects of Recalcitrance toward Antibiotics. Microbiol. Mol. Biol. Rev..

[46] Asma S.T., Imre K., Morar A., Herman V., Acaroz U., Mukhtar H., Arslan-Acaroz D., Shah S.R.A., Gerlach R. (2022). An Overview of Biofilm Formation–Combating Strategies and Mechanisms of Action of Antibiofilm Agents. Life.

[47] Lucero M., Estell R., Tellez M., Fredrickson E. (2009). A Retention Index Calculator Simplifies Identification of Plant Volatile Organic Compounds. Phytochem. Anal..

[48] Gaur P., Hada V., Rath R.S., Mohanty A., Singh P., Rukadikar A. (2023). Interpretation of antimicrobial susceptibility testing using European Committee on Antimicrobial Susceptibility Testing (EUCAST) and Clinical and Laboratory Standards Institute (CLSI) breakpoints: Analysis of agreement. Cureus.

[49] Sabaeifard P., Abdi-Ali A., Soudi M.R., Dinarvand R. (2014). Optimization of Tetrazolium Salt Assay for *Pseudomonas aeruginosa* Biofilm Using Microtiter Plate Method. J. Microbiol. Methods.

[50] Kim S., Kim M.J., Kang H.Y., Seol S.Y., Cho D.T., Kim J. (2010). A Simple Colorimetric Method for Testing Antimicrobial Susceptibility of Biofilmed Bacteria. J. Microbiol..

