# Supplementary material for: Antibiofilm and Antimicrobial Potentials of Novel Synthesized Sulfur Camphor Derivatives
Source: Int J Mol Sci. 2024 Oct 10;25(20):10895. doi: 10.3390/ijms252010895 (PMC11507198; doi:10.3390/ijms252010895)
Supplement: Supplementary file 1 [file ijms-25-10895-s001.zip › ijms-3157581-supplementary.pdf]

Supplementary Materials

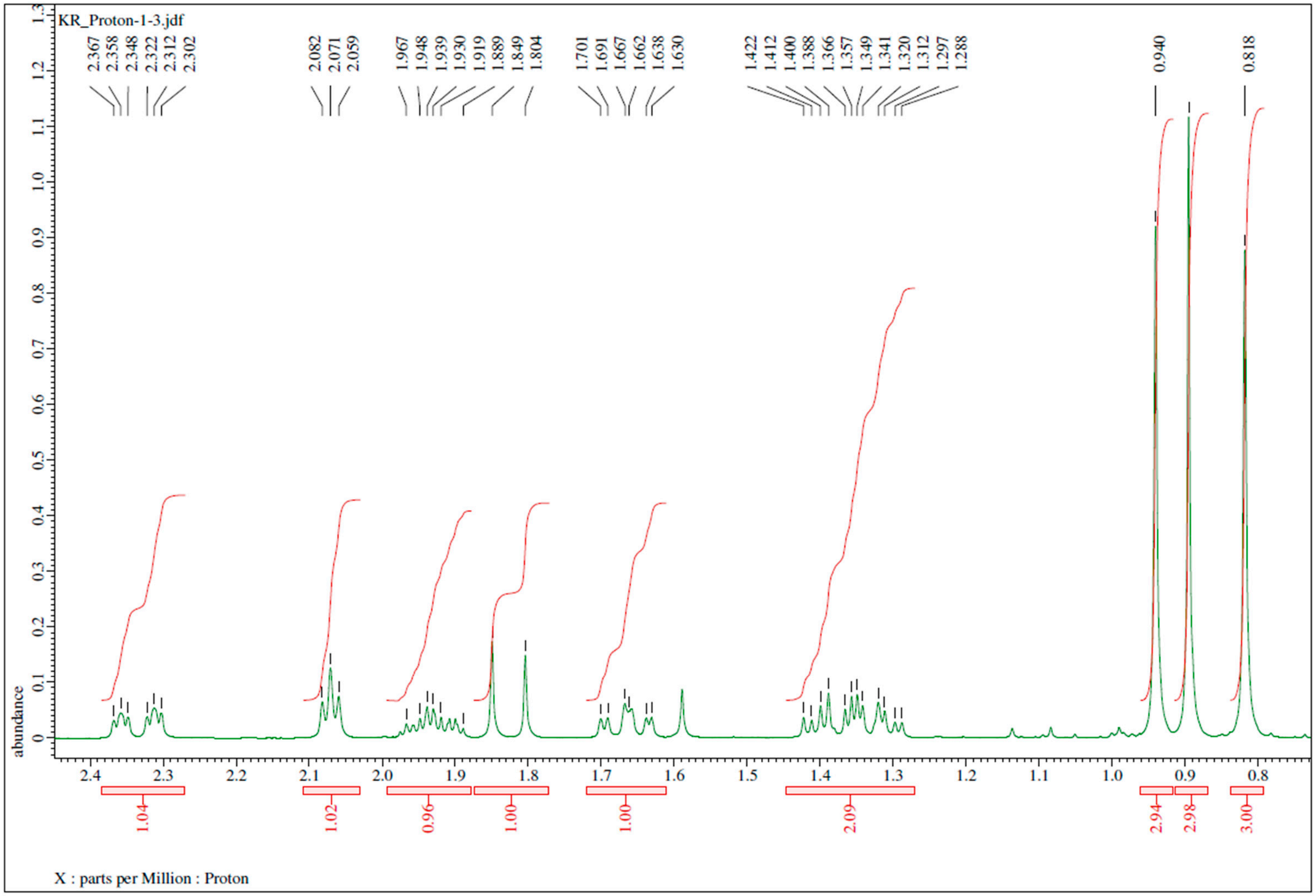

Figure S1. ^{1}H NMR (400 MHz, $CDCl_3$) spectrum of *rac*-camphor

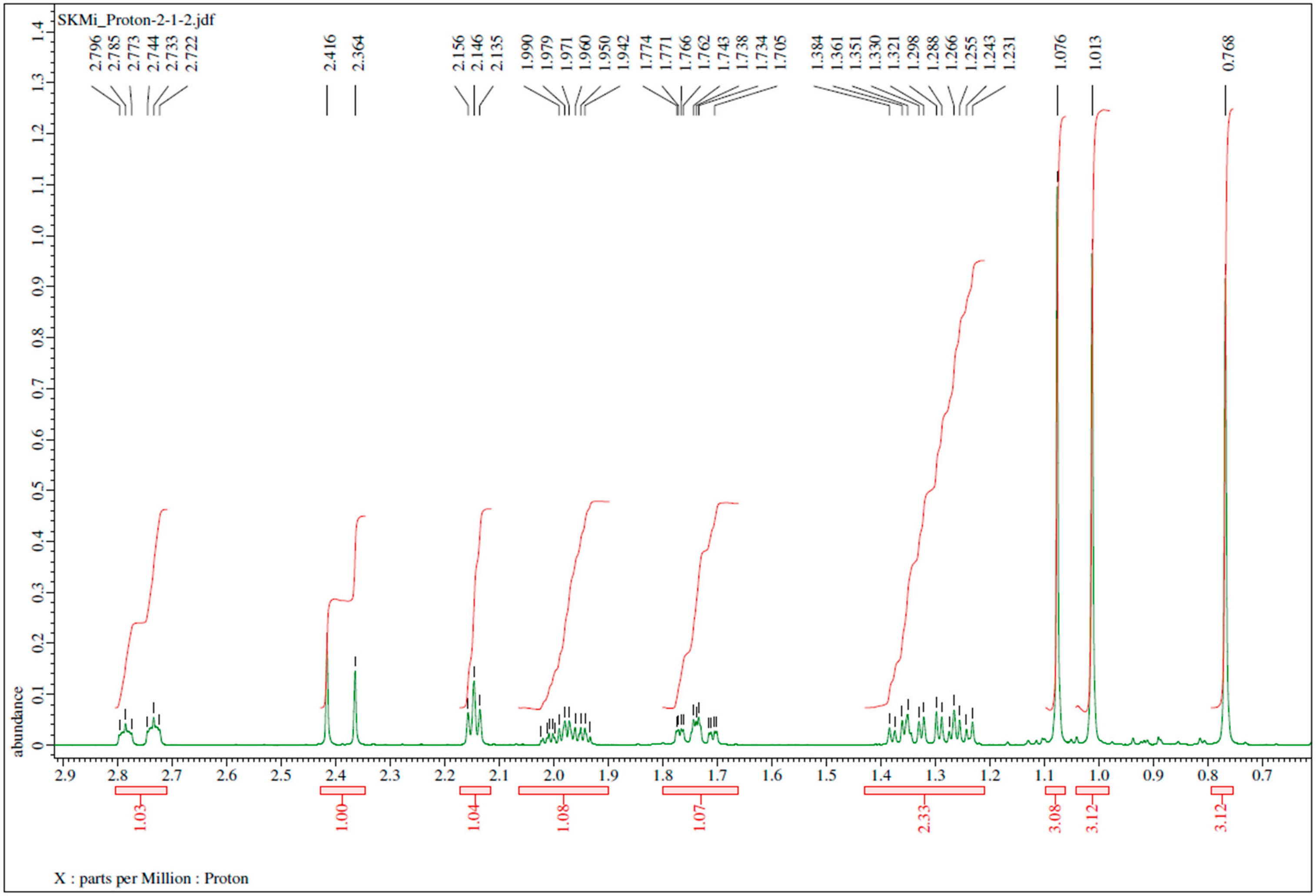

Figure S2. ^{1}H NMR (400 MHz, $CDCl_3$) spectrum of *rac*-thiocamphor

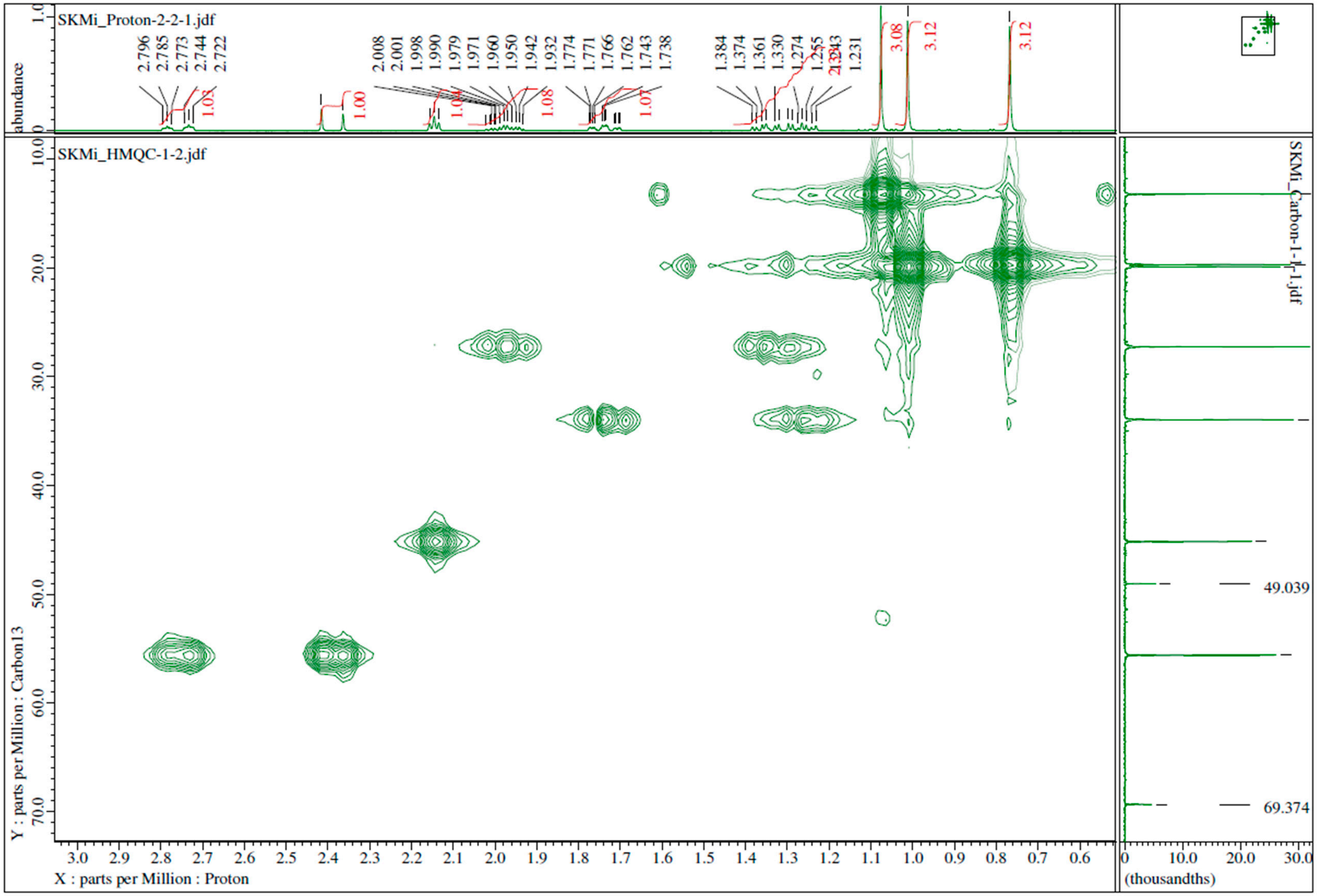

Figure S3. HMQC (100 MHz, $CDCl_3$) spectrum of *rac*-thiocamphor

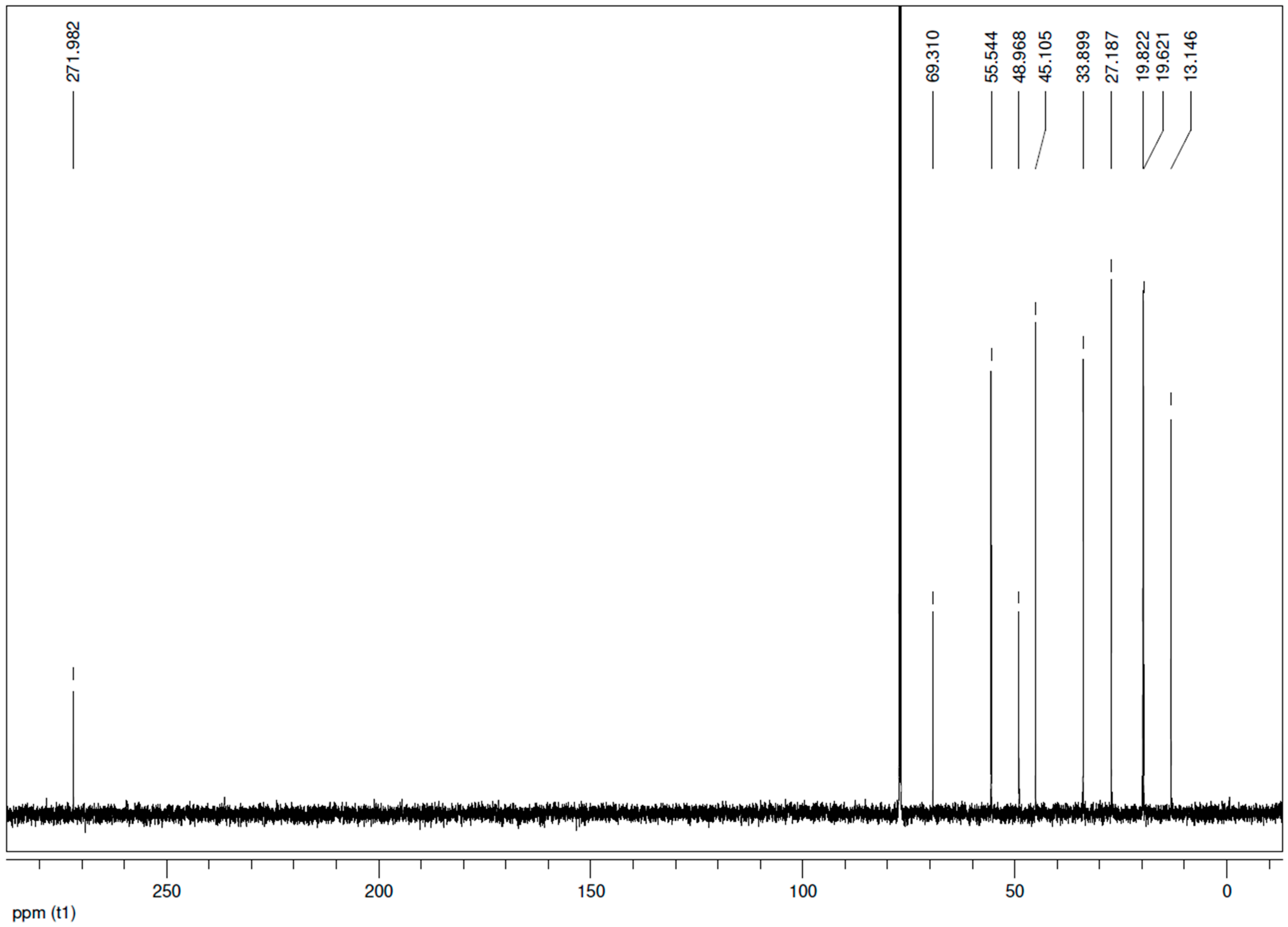

Figure S4. ^{13}C NMR (151 MHz, $CDCl_3$) spectrum of *rac*-thiocamphor

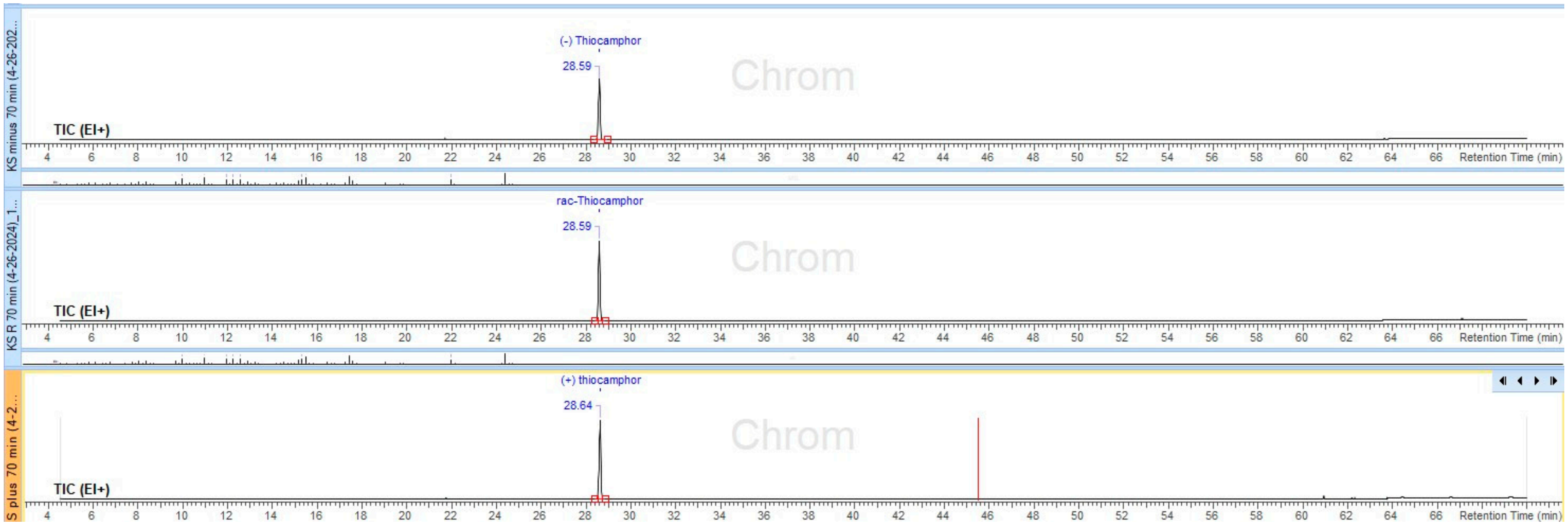

Figure S5. Chromatograms of racemic and isomers thiocamphor

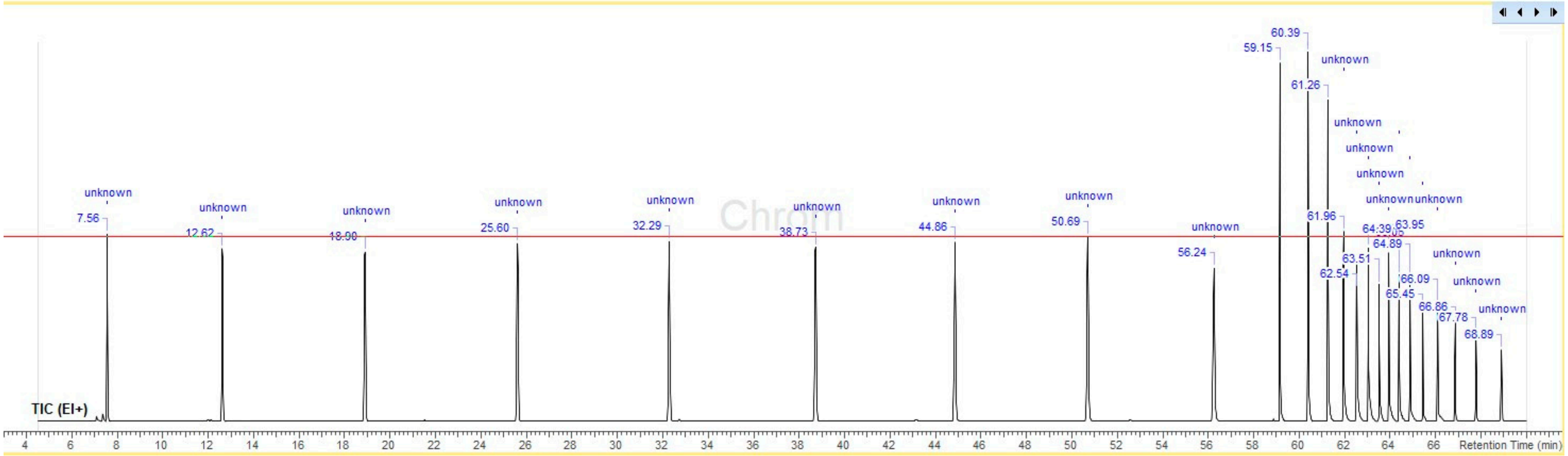

Figure S6. Chromatogram of *n*-alkanes, used for retention index calculations

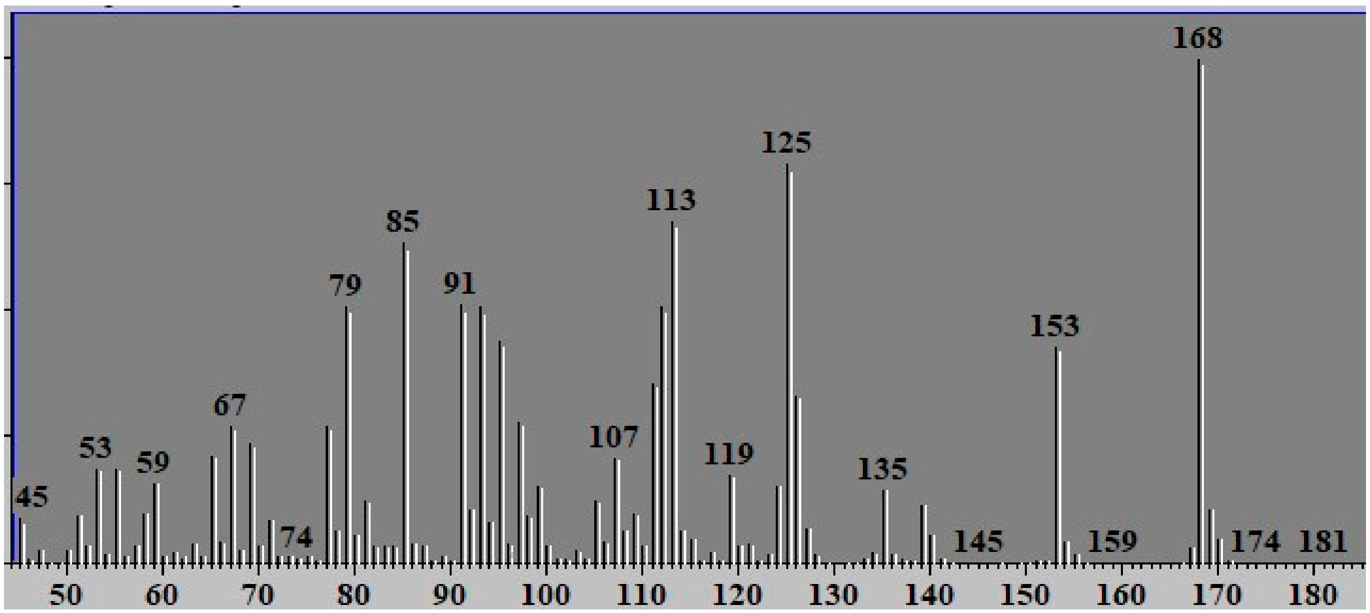

Figure S7. Experimental mass spectrum of thiocamphor

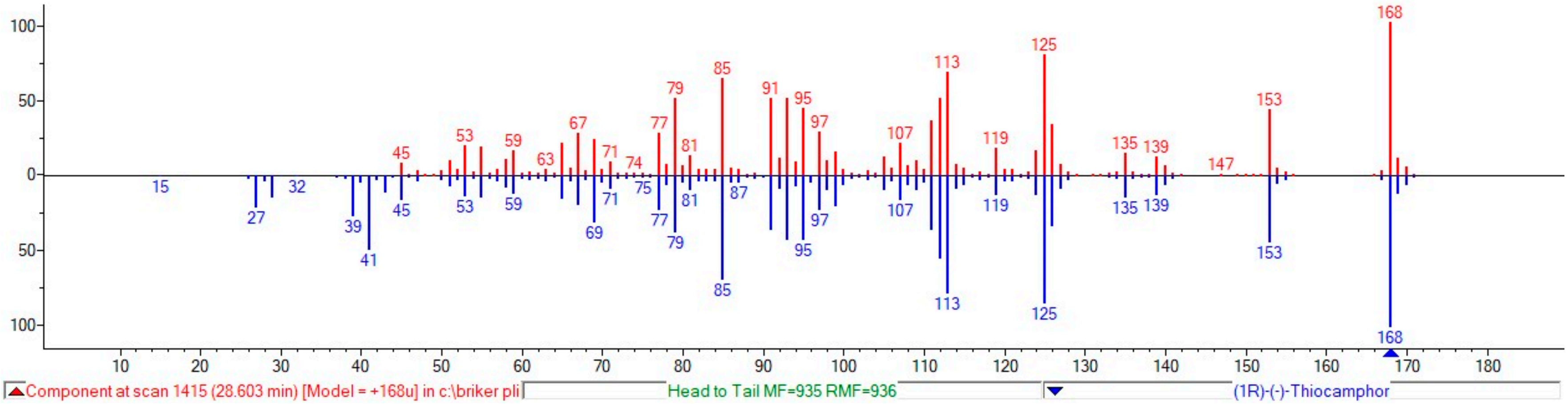

Figure S8. Comparison of theoretical mass spectra with the spectrum of R, R-(-)-thiocamphor

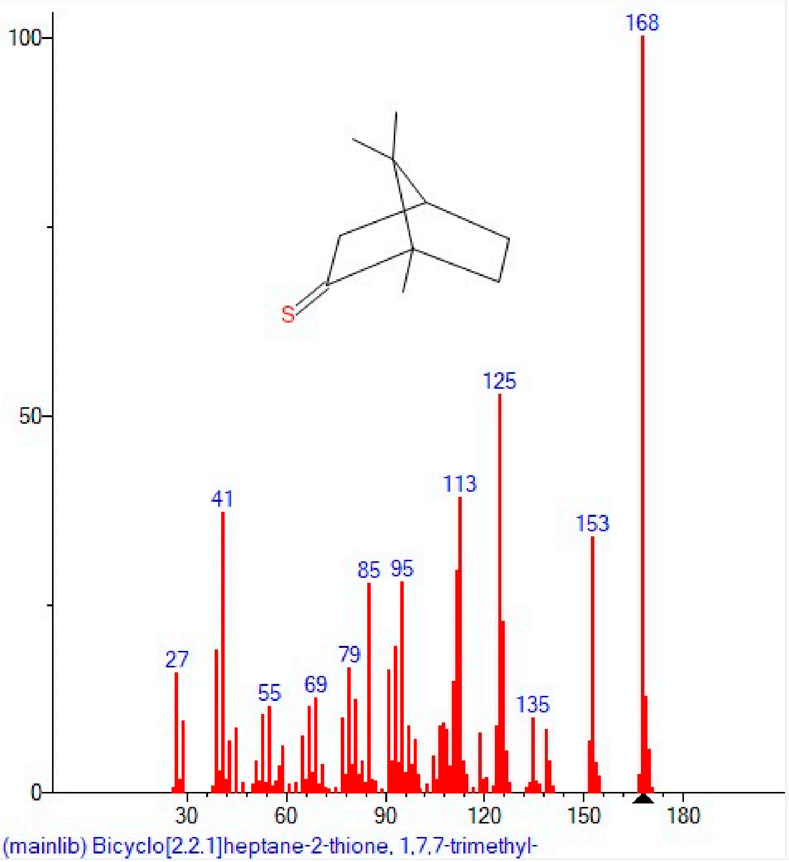

Name: Bicyclo[2.2.1]heptane-2-thione, 1,7,7-trimethyl-
Formula: $C_{10}H_{16}S$
MW: 168 Exact Mass: 168.097272 CAS#: 7519-74-6 NIST#: 158259 ID#: 218516 DB: mainlib
Contributor: Chemical Concepts
InChIKey: AAADKYXUTOBAGS-UHFFFAOYSA-N Non-stereo
Synonyms:
1.2-Bornanethione
2.Thiocamphor
3.1,7,7-Trimethylbicyclo[2.2.1]heptane-2-thione #
Other DBs:
-----Drug-----
DrugBank
-----Environmental-----
EPA Chemicals & Products; EU Suspected Contaminants
-----Metabolite-----
PDB

Estimated semi-standard non-polar retention index (n-alkane scale):
Value: 1258 iu
Confidence interval (Sulfur-containing): 46(50%) 198(95%) iu

AI predicted semi-standard non-polar retention index (n-alkane scale):
Value: 1298 iu
Confidence interval : 25(50%) 80(95%) iu

Figure S9. Mass spectrum of thiocamphor from the NIST23 database

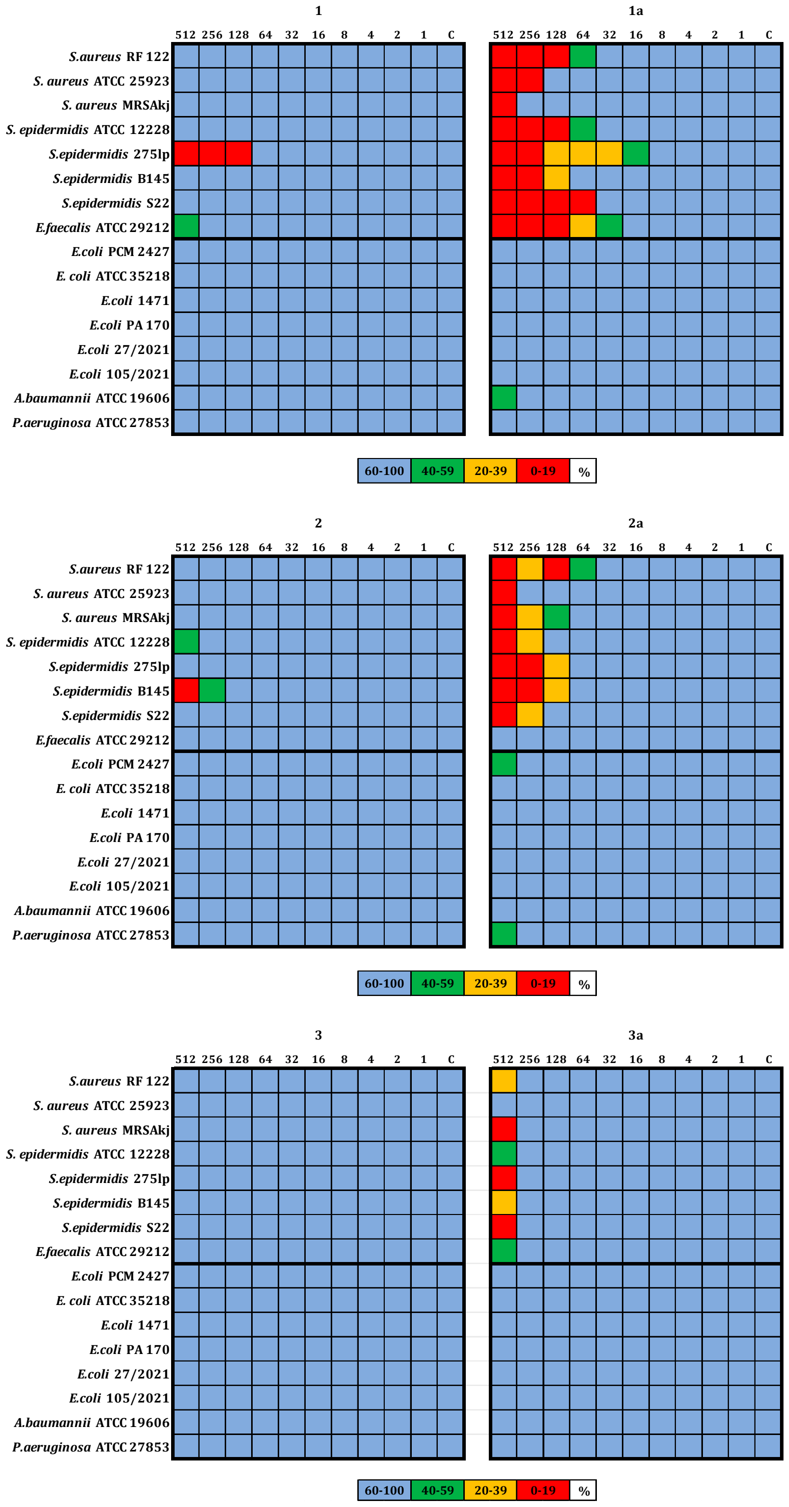

Figure S10. The activity of sulfur derivatives of camphor on the tested Gram-positive and Gram-negative strains presented as a heatmap. The following numbers: 1, 2, 4, 8, 16, 32, 64, 128, 256, and 512 in the graphs indicate the concentrations of tested compounds (1, 1a, 2, 2a, 3, 3a) expressed in µg/mL.

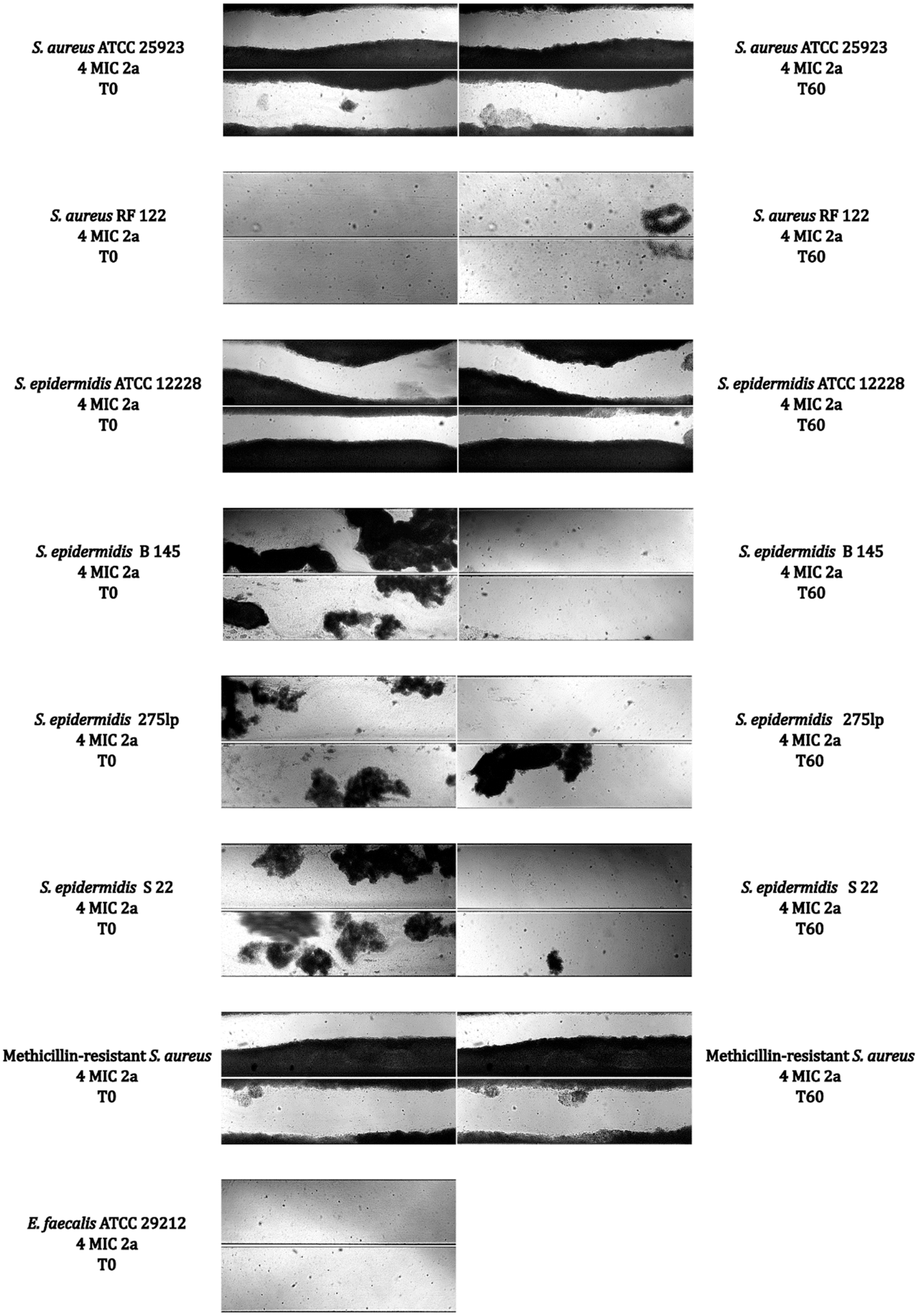

Figure S11. Representative Bioflux photographs showing the effect of the studied sulfur derivatives of camphor on the 24-hour biofilm formed by the tested Gram-positive strains
